# Prevention of suicidal behaviour: Results of a controlled community-based intervention study in four European countries

**DOI:** 10.1371/journal.pone.0224602

**Published:** 2019-11-11

**Authors:** Ulrich Hegerl, Margaret Maxwell, Fiona Harris, Nicole Koburger, Roland Mergl, András Székely, Ella Arensman, Chantal Van Audenhove, Celine Larkin, Mónika Ditta Toth, Sónia Quintão, Airi Värnik, Axel Genz, Marco Sarchiapone, David McDaid, Armin Schmidtke, György Purebl, James C. Coyne, Ricardo Gusmão, on behalf of The OSPI-Europe Consortium

**Affiliations:** 1 Department of Psychiatry, Psychosomatics, and Psychotherapy, Goethe-Universität Frankfurt, Frankfurt, Germany; 2 Nursing, Midwifery and Allied Health Professions Research Unit, University of Stirling, Stirling, Scotland, United Kingdom; 3 Department of Research Services, University of Leipzig, Leipzig, Saxonia, Germany; 4 Department of Psychology, Bundeswehr University Munich; 5 Institute of Behavioral Sciences, Semmelweis University Budapest, Budapest, Hungary; 6 National Suicide Research Foundation and School of Public Health, University College Cork, Cork, Ireland; 7 Center for care research and consultancy at KU Leuven (LUCAS), University of Leuven, Leuven, Belgium; 8 CEDOC, Faculdade de Ciências Médicas, Universidade Nova de Lisboa, Lisbon, Portugal; 9 Estonian-Swedish Mental Health and Suicidology Institute (ERSI), Tallinn, Estonia & Tallinn University, Tallinn, Estonia; 10 Department of Psychiatry, Psychotherapy and Psychosomatic Medicine, Otto-von-Guericke University, Magdeburg, Saxonia-Anhalt, Germany; 11 Department of Medicine and Health Sciences, University of Molise, Campobasso, Italy; 12 London School of Economics and Political Science, London, United Kingdom; 13 Department of Psychiatry, Psychosomatics and Psychotherapy, University of Wuerzburg, Wuerzburg, Bavaria, Germany; 14 Perelman School of Medicine, University of Pennsylvania, Philadelphia, Pennsylvania, United States of America; 15 ISPUP, Instituto de Saúde Pública da Universidade do Porto, Porto, Portugal; Harvard University, UNITED STATES

## Abstract

The ‘European Alliance Against Depression’ community-based intervention approach simultaneously targets depression and suicidal behaviour by a multifaceted community based intervention and has been implemented in more than 115 regions worldwide. The two main aims of the European Union funded project “Optimizing Suicide Prevention Programmes and Their Implementation in Europe” were to optimise this approach and to evaluate its implementation and impact. This paper reports on the primary outcome of the intervention (the number of completed and attempted suicides combined as ‘suicidal acts’) and on results concerning process evaluation analysis. Interventions were implemented in four European cities in Germany, Hungary, Portugal and Ireland, with matched control sites. The intervention comprised activities with predefined minimal intensity at four levels: training of primary care providers, a public awareness campaign, training of community facilitators, support for patients and their relatives. Changes in frequency of suicidal acts with respect to a one-year baseline in the four intervention regions were compared to those in the four control regions (chi-square tests). The decrease in suicidal acts compared to baseline in the intervention regions (-58 cases, -3.26%) did not differ significantly (χ^2^ = 0.13; p = 0.72) from the decrease in the control regions (-18 cases, -1.40%). However, intervention effects differed between countries (χ^2^ = 8.59; p = 0.04), with significant effects on suicidal acts in Portugal (χ^2^ = 4.82; p = 0.03). The interviews and observations explored local circumstances in each site throughout the study. Hypothesised mechanisms of action for successful implementation were observed and drivers for ‘added-value’ were identified: local partnership working and ‘in-kind’ contributions; an approach which valued existing partnership strengths; and synergies operating across intervention levels. It can be assumed that significant events during the implementation phase had a certain impact on the observed outcomes. However, this impact was, of course, not proven.

## Introduction

Completed and attempted suicides continue to be a significant mental and public health issue. In 2015, nearly 800,000 people worldwide died by suicide, 58,000 of them in Europe; and the number of attempted suicides is estimated to be more than 20 times higher than this [1]. Suicidal behaviour is often related to mental illness, with depressive disorders being most important in this context [1,2]. Around 30 million European citizens suffer from unipolar depression per year [3], most of them with no or suboptimal treatment. Improving the care of people with depression is therefore a central element in suicide prevention strategies [4].

Many factors at the level of the patient, the health care systems and the society contribute to both the gaps in the care for people with depression and the high rate of suicidal behaviour. For example, at the level of the depressed and /or suicidal patients, shame or fear regarding stigmatization have a negative impact on help seeking behaviour and on reporting mental symptoms or suicidal tendencies (e.g., [5–7]). Lack of expertise in exploring, diagnosing and treating depression and suicidal tendencies at the level of general practitioners and the difficulties to get rapid access to specialized care are other relevant factors (e.g., [8,9]). At the level of the society, misconceptions in the general population about depression and suicidal behaviour together with the stigmatisation of people with mental illnesses contribute to the mentioned gaps in care [10–12].

Evidence based suicide preventive single measures have been identified and recently reviewed [4,13]. However, in view of the many factors associated with suicidal acts, interventions which combine several of these single measures are most promising and are recommended by the WHO as strategy for suicide prevention [13,14]. Combining single measures has been shown to entail not only additive suicide preventive effects, but also synergistic and catalytic effects [15,16]).

Published results of such multifaceted interventions targeting suicide have been recently summarized [13]. Within controlled designs, preventive effects on suicidal behaviour were observed in several [17–23] but not in all of the studies [24]). Differences concerning the design, the intensity, the elements and the size of the interventions make it difficult to explain the reasons for differences in efficacy of these multifaceted interventions.

Consistent evidence provided by several controlled studies is available for the preventive effects concerning suicidal behaviour of the 4-level intervention strategy promoted by the European Alliance against Depression (www.eaad.net, [20–23]). It comprises the following four intervention levels:

1. general practitioners (GPs). Interventions at this level are important because most patients with depression are seen at the primary care level. Considerable diagnostic and therapeutic deficits concerning both depression and suicidality have been documented at the level of GPs and improving this situation is likely to have a positive impact on both the burden associated with depression and the rate of suicidal acts [4,14,25,26]
2. The general public. Lack of knowledge about treatment and stigma related to both depression and suicide have negative consequences on help seeking behaviour and increase the emotional burden for those affected. Public awareness campaigns are a strategy to address these issues as indicated also by surveys on public attitudes run within OSPI-Europe (“Optimising Suicide Prevention programmes and their Implementation in Europe” funded by the European Union (EU), 7th Framework Programme; see data from the OSPI-project [27–29], see also [4]).
3. Community facilitators and gatekeepers. In addition to the general public there are professional groups such as priests, policemen, pharmacists which are in close contact with people with mental health problems and suicidal tendencies. To improve health literacy in these groups is likely to contribute to the reduction of therapeutic deficits (concerning attitudes and knowledge about depression and suicides in the regions studied within OSPI-Europe; see [30–34]). Also journalists are an important target group in order to avoid unfavourable media coverage concerning suicide, which has the risk to induce copycat suicides, the so called Werther effect [1].
4. Patients, their relatives and high-risk groups such as survivors of a suicide attempt. Interventions at this level aim at improving knowledge, help seeking behaviour and self-help activities.

Using this 4-level intervention approach, preventive effects on suicidal behaviour were first shown with a model project in Nuremberg. A significant reduction of suicidal acts (- 24%, primary outcome) was observed during the 2-years intervention in Nuremberg (480.000 inhabitants) compared to both the baseline year and the control region (Wuerzburg, 270,000 inhabitants). This effect turned out to be sustainable [20,21]. Further evidence for this approach has been obtained from evaluations in other regions in Germany and Hungary [22,23,35]. The community based 4-level intervention has been implemented in the meanwhile in more than 115 regions in Germany and other countries in and outside of Europe (www.eaad.net) (reviewed in [35]). This suggested that the 4-level intervention showed promise and that it was worthy of a large-scale evaluation.

The 4-level intervention concept, promoted by EAAD, and the broad experiences with it´s regional implementations have been the basis for the research project OSPI-Europe.

Such complex community based interventions can be influenced concerning both the implementation process and its effects by a variety of circumstantial factors or unforeseeable events. Within OSPI-Europe contextual information on such factors was systematically collected during the intervention period and they have been shown to play an important role in either facilitating or impeding opportunities for synergies between levels of this intervention [16]) (e.g., an unforeseeable natural disaster or a national election interfering with a public campaign and impeding the organization of train-the-trainer seminars). The corresponding information can be gathered via regular evaluation of the content of regional press. Such knowledge can help to fill the ‘implementation gap’ which outcomes based studies fail to capture but which is crucial for replication of interventions, for interpreting and understanding outcomes, and for improving implementation science [36,37]. The process evaluation within OSPI-Euope was informed by the realist evaluation approach (REF) [38] and we took the innovative step of embedding this approach within a controlled study, adding an important interpretive dimension to the primary and secondary outcome measurement.

Aim of this paper is to report the effects of the 4-level interventions in four intervention compared to four control regions on suicidal acts (addition of completed and attempted suicides, primary outcome) and to contextualise these findings based on an independent and prospective process analysis and assessment of intervening context factors. Results for several secondary outcomes such as public mental health literacy, and partly concerning process evaluation findings on implementation, have been published elsewhere [15,28–31].

## Materials and methods

### The OSPI-Europe intervention

OSPI interventions were building on the 4-level intervention concept from the Nuremberg Alliance against Depression and were implemented in four regions with matched control regions in Germany, Hungary, Portugal and Ireland. Informed by systematic literature review concerning evidence based suicide preventive measures [39] access to lethal means was added as a new intervention element (to identify and secure hotspots concerning suicides in intervention regions, to restrict package size of certain drugs by cooperating with pharmacists and GPs, see [4,40]).

OSPI-Europe was a complex 4-level suicide prevention programme, targeting professionals, the public, patients, and multiple organizations across the health, social, education and judicial/policing sector (for details see [41]).

The aim of OSPI-Europe was „to provide diverse regional policy makers and the European Commission with an evidence based, efficient concept for suicide prevention along with the corresponding materials and instruments for the multifaceted intervention and guidelines for the implementation process”([41], page 2).

The primary outcome was the number of suicidal acts (completed and attempted suicides combined). Among others, secondary outcomes included changes in knowledge, attitudes and awareness of suicide and depression for: GPs (Level 1); the public (Level 2); and Community Facilitators (CFs ie community-based professionals such as teachers, social workers and police force, Level 3) (for details see [27–31,41].

### Sample

In each of the four selected European countries in this analysis (Ireland, Portugal, Germany and Hungary) an intervention and control region was selected based on population size (having at least 150,000 inhabitants in each geographical area; see Table 1); regional interest in hosting the OSPI-Europe interventions; and that no previous suicide prevention or depression awareness programme had taken place in those regions. For this purpose, cooperating OSPI-Europe project partners in Germany, Hungary, Ireland and Portugal had been asked.

**Table 1:** OSPI-Europe intervention and control populations (2008).

	Intervention Region	Control Region
**Germany**	Leipzig 515,469	Magdeburg 230,047
**Hungary**	Miskolc 170,234	Szeged 169,030
**Ireland**	Limerick 188,299	Galway 237,898
**Portugal**	Amadora 172,110	Almada 166,103
**Total populations**	**1,046,112**	**803,078**

Regarding Amadora and Almada, they are two very similar suburban to Lisbon councils, with approximately the same population and demographic structure and separated by the river Tagus which would reduce contamination. The psychiatric care organization—hospitals, number of beds, patients—and primary care professional to users ratio were also very similar, with slightly more staff in Amadora. Moreover, there were good relations with the leaders of care organizations. In addition, Amadora is very densely populated by square meters of area which made it well suited for public campaigns.

Regarding the Hungarian intervention region, Miskolc is a city in North-Eastern Hungary, with a population close to 170,000 inhabitants (in 2010). Miskolc is the fourth largest city of Hungary (behind Budapest, Debrecen and Szeged; the second-largest city with agglomeration). It is also the county capital of Borsod-Abaúj-Zemplén and the regional centre of Northern Hungary. Miskolc had a strong economy going back hundreds of years. Its heavy industry was very strong in the second half of the 20^th^ century but the collapse of the socialist system and the following recession caused a strong recession and most of the industrial companies had been closed. Thus, the unemployment rate went up and many citizens have left the city. The portion of the Roma population is high in the city, as it is in the whole region.

The control region, Szeged is the third largest city of Hungary, the largest city and regional centre of the Southern Great Plain and the county town of Csongrád county. The University of Szeged is one of the most distinguished universities in Hungary. Moreover, Szeged is one of the centres of the food industry in Hungary. The county and the whole region are surrounded with small farms. The region has also a high unemployment rate due to the recession in the agricultural sector. This region is famous of the very high suicide rate going back for a long time.

The main reasons for the selection of these two cities were as follows:

1. Both cities were large enough to collect reliable data, and also in both cities there were large hospitals.
2. Both cities were county centres, with high unemployment rates, but with a bit different background.
3. The infrastructure was similar.
4. There were good relations with the leaders of the psychiatric departments and with the hospitals.

A random selection of intervention regions from all member states of the European Union (EU) was not performed in view of “multiple factors on which representativeness could be called in question” ([41]; page 4). Instead, intervention regions (and the corresponding control regions) were selected in four EU member states (Ireland, Germany, Hungary, Portugal) representing quite different health systems (like a tax funded public health service in Ireland and a centralised national health insurance fund in Hungary) and different cultural characteristics [41].

Figures for Amadora and Almada are based on data from the Statistical Office of Portugal, figures for Leipzig on data from the Leipzig Information Service, figures for Magdeburg on data from the Statistical Office of Saxonia-Anhalt, figures for Miskolc and Szeged from the Hungarian Statistical Office. Figures for Limerick and Galway were based on regional estimates because exact figures for 2008 were not available at the time.

The study compared a one-year baseline period to a two-year index time period after inception of the intervention. Comparisons were made with both the baseline period and with the control region. Thus, pre-post differences for the outcomes could be assessed for each region and by using a difference-in-differences approach it was possible to compare intervention and control regions regarding changes in the frequency of suicidal acts. Required and optional intervention activities were pre-defined and conducted for a minimum of 1.5 years. Table 2 shows the intensity of mandatory activities for intervention levels 1 to 3. Several optional actions were undertaken depending on local needs and circumstances (see S8 Table). Level 4 activities included supporting self-help groups. Efforts to restrict access to lethal means included the local identification and security inspection of locations where people frequently take their lives.

**Table 2:** Overview of the OSPI-Europe intervention activities run in the four intervention regions.

	Leipzig (Germany) population 515,469	Miskolc (Hungary) population 170,234	Limerick (Ireland) population 188,299	Amadora (Portugal) population 172,110	Total
**Level 1 Primary care training**					
General Practitioners	86	50	96	68	302
**Level 2 Public awareness campaign**					
Flyers	175,200	60,000	40,000	130,000	405,200
Posters (including optional sizes)	2,748	3,303	10,025	5,045	21,121
Public events	45	9	1	8	63
**Level 3 Community facilitator (CF) training**					
Pharmacists	51	50	15	46	162
Priests and religious leaders	36	53	37	23	149
Police officers	134	13	494	302	943
Total CF (including optional target groups)	915	355	631	1,509	3,410

Overall, the intervention addressed suicide prevention through measures aiming at reducing the diagnostic and therapeutic deficits regarding depression and suicidal tendencies. The therapeutic deficits were defined as deviations from the available recommendations of the respective national guidelines for the treatment of depression [42,43]. Elements were supporting improved mental health literacy in the general public as well as training in recognising and dealing with suicidal behaviour. The intervention also included strategically placed emergency cards containing information of where to seek help if one has suicidal thoughts (distributed in practices of general practitioners and other physicians), which were also offered to people after a suicide attempt who were treated in a psychiatric hospital.

In Amadora, emergency cards were level 4 offers for preselected subgroups (see below) and were distributed in Accident and Emergency hospitals for self-harmers, their friends and family within a perspective of indicated prevention for at risk identified populations. There were no other special offers at this level.

In Miskolc, flyers were produced in the beginning providing information about depression and treatment possibilities. The emergency cards could be taken out of the flyer and held separately together with other cards. Places of their dissemination were offices of general practitioners, pharmacies, health centers, social institutions, hospitals, schools, libraries, supermarkets and community centres.

The local OSPI teams also considered access to lethal means by identifying suicide ‘hotspots’ and worked with local authorities to support suicide prevention measures. However, the latter often involved changes to infrastructure (such as barriers on bridges) that involved time consuming burocratic decision processes which were not finished within the time frame of OSPI-Europe.

Fidelity to the intervention and implementation strategies was measured by the independent process evaluating team (see below) by using checklists for the intervention and implementation strategies.

### Outcome measures and data assessment

#### Outcomes

The *main outcome* consisted of *suicidal acts*: the sum of completed suicides and attempted suicides.

Whereas *completed suicides* were defined according to the ICD-10 codes [44] for intentional self-harm as an external cause of morbidity and mortality (X60-X84), *attempted suicide* was defined as “an act with non-fatal outcome, in which an individual deliberately initiates a non-habitual behaviour that, without intervention from others, will cause self-harm, or deliberately ingests a substance in excess of the prescribed or generally recognised therapeutic dosage, and which is aimed at realizing changes which the subject desired via the actual or expected physical consequences” [45]. Repeated suicide attempts conducted by the same persons during the study period were not excluded because the focus of analyses were the number of suicidal acts.

Habitual self-harm without suicidal intent is excluded by this definition. Data on attempted suicides were collected for the intervention as well as the control regions together with a given set of core variables (e.g. age, gender, region, suicide method). A standardised questionnaire for the assessment of data and a codebook containing the variables for the registration of suicide attempts were used by all partners to ensure comparability in data acquisition. Unclear cases which could not instantly be classified as a suicidal act or any other behaviour, for example habitual deliberate self-harm, were pooled. These cases were then categorised by an internal expert who was blinded regarding the timepoint and region of the event.

The procedures for the assessment of attempted suicides differed between countries, but care was taken to assure consistency of data assessment procedures over time. In Hungary and Portugal and two of four German centres, all admissions to hospitals because of suicide attempts were assessed by using retrospective analysis of patient records. In Germany, in two further participating centres the data were assessed following a prospective design via personal interviews by trained staff. In Ireland, data are routinely collected on patients presenting to a hospital with a suicide attempt using standard methods for case ascertainment and definition, processed in the National Self-Harm Registry Ireland (NSHRI). Levels of agreement between the data registration officers in terms of case ascertainment were high (Kappa = 0.97) [46].

*Highly lethal suicide attempts* represented another outcome and implied the exclusion of the lower-risk suicide methods “Intentional drug overdose” and “use of sharp objects” which could be shown to have low case fatality ratios (1.8%) [47].

Thus, more lethal suicide attempts included the following suicide methods: Hanging (X70), drowning (X71), firearms (X72-X75), jumping (X80), moving objects (X81,X82), other suicide methods (X76, X77, X79, X83, X84).

If several methods were applied, the most lethal suicide method according to previous findings [48] was classified as the primary suicide method and entered into subsequent analyses.

**Process and context evaluation.** The Process Evaluation was informed by the UK’s Medical Research Council’s framework for conducting and reporting process evaluation studies [49]. This framework sets out the key functions of a process evaluation as well as the relations among them and the key components of a process evaluation, which are: context (C); implementation (I); and mechanisms of action (M); impacting on outcomes (O) (CIMO). This process evaluation was informed by the realist evaluation approach (REF) [38] drawing on longitudinal case studies, where ‘cases’ were constituted by the intervention regions.

**Data collection.** Data collection was coordinated and analysed by the Process Evaluation Team (PET) who were not involved in any implementation activity. Data collection involved progress tracking questionnaires, qualitative interviews / focus groups conducted in each of the four intervention regions at six monthly intervals and participant observation conducted by the process evaluation researcher (FH) at our six monthly OSPI-Europe meetings. The process evaluation team consisted of three researchers with extensive qualitative health services research experience, with doctorates in social anthropology (FH), sociology (MM) and psychology (ROC). Interviews and focus groups were conducted by experienced qualitative researchers, three of whom had Masters in psychology, and the fourth being an academic GP. All four had some involvement in implementation activity, which, although not ideal, nevertheless resulted in good quality data that included critical reflections of OSPI-EUROPE. This enabled us to explore both barriers and facilitators to implementation.

Progress tracking questionnaires were completed for each intervention region to capture details of occupational groups receiving training, public campaign events and activities (e.g distribution of flyers/posters) and any support offered to patients/families. The PET developed the semi-structured interview and focus group topic guides and they provided training in using these instruments to the local OSPI-Europe researchers who were conducting the data collection in participants’ first language. Interviews ranged in duration from 30–50 minutes and focus groups were 40–60 minutes. These took place in settings of most convenience to participants, including their workplaces or university premises.

Interview and focus group participants consisted of: members of local Advisory Groups representing a range of local organizations with an interest or remit for mental health and/or suicide prevention which were set up to facilitate local implementation; or recruited via local stakeholders who were considered as necessary partners for implementation (e.g. primary care practitioners, pharmacists, local authority partners). The PET assisted local OSPI teams to identify individuals and organizations they considered to be instrumental for local implementation. The qualitative data collection explored, among other issues, barriers and facilitators to implementation and the contexts of implementation. Contextual data included exploring local capacity issues for implementing the intervention, regional economic change and local/national mental health policy landscapes.

The process evaluation researcher also conducted participant observation at OSPI-Europe project meetings, in order to follow up questions with implementation team researchers and observe presentations from each region’s lead. These observations were recorded as fieldnotes. Stakeholder workshops were conducted in each region at the end of the implementation period to reflect on capacity and sustainability. Two further workshops were held with the whole OSPI-Europe team in order to explore and discuss lessons learned from implementation. Table 3 summarises the data collected at each site.

**Table 3:** Summary of data collection and sources.

	Interviews	Focus Groups	Workshops	Questionnaires
**Germany**	14	4	1	5
**Hungary**	10	4	1	5
**Ireland**	13	3	1	5
**Portugal**	10	1	1	5

There had been six meetings‘ fieldnotes at implementation team meetings, one synergistic effects workshop (focus: work package leads and intervention site researchers), five workshops for the optimization of the 4-level approach and three focus groups (focus: all OSPI-EUROPE leads and researchers). In total, 47 interviews, 15 focus groups, six meetings‘ observations/fieldnotes and six workshops had been performed.

Audio-recorded interviews and focus groups were transcribed verbatim and translated into English (where necessary) for analysis. The full methods of the OSPI-Europe process and context evaluation have been reported elsewhere, including details of participants and ethical approval obtained in each region [15].

**Data analysis.** Analysis of process evaluation data was facilitated by the Framework Approach [50], using NVivo (V9) software to store and organize the data for analysis. Three members of the PET (FH, MM, RO’C) read transcripts to identify contextual and implementation issues, mechanisms of change, and events or actions identified by key stakeholders as likely to impact on intervention delivery and outcomes. These were discussed, compared across sites and agreed. The interview, focus group and observational data were charted under thematic headings for each country, with each intervention site representing the unit of analysis for the case studies. A framework was developed to explore the barriers and facilitators to implementation. Both within-case and cross-case themes were identified via the framework method, which were then developed further using an interpretive approach. The findings presented in this paper are the results of a meta-narrative of the overall case studies: a necessary mechanism to convey the key information for each case study in relation to CIMOs and for each level of intervention. Presenting this complex meta-narrative has been at the expense of presenting numerous examples of data extracts as evidence of findings, but is nevertheless built on these data.

#### Statistical analysis of outcomes data

Effect sizes from the Nuremberg Alliance against Depression [20] informed the sample size calculation prior to initiating the OSPI-Europe intervention. With the level of significance (α) set at 0.10 (two-tailed testing) and the required power (1-β) set at 0.80, assuming a decrease in suicidal acts compared to baseline of 24.8% in the intervention region and of 0 in the control region, a population of 119,071 subjects in the intervention and the control regions of each country would be necessary to observe statistically significant change.

For calculations, the absolute numbers of suicidal acts were preferred over rates because they are more informative and because demographic data revealed that there were only minor differences in the changes in the number of the population in the intervention and corresponding control regions between 2008 and 2011 (Germany: delta = -0.56%; Hungary: delta = 0.96%; Ireland: delta = 3.50%; Portugal: delta = 3.01%). Population change in the OSPI intervention and control regions for the years 2008–2011 (stratified for gender) are summarized in S1 Table, the corresponding differences between OSPI intervention and control regions in S2 Table. These minor changes were corrected by a loading factor adjusting for changes of gender-specific population figures in the intervention regions (see S3 Table). Absolute numbers also reflect better the differences in the size of populations in the different cities. In addition, rates are not comparable between the cities because they differed concerning the completeness with which suicide attempts were assessed.

Concerning the primary outcome χ^2^ tests for two-by-two tables, with the row variable being “region” and the column variable being “time” (1 = baseline; 2 = arithmetic means for the two years after onset of the awareness campaign) were calculated.

Moreover, comparison of changes in the frequency of suicidal acts in the intervention versus control region was performed by using the Cochran-Mantel-Haenszel procedure, with the variable “region” being used as a stratification variable. Homogeneity of odds ratios across countries was tested by using the Breslow-Day test.

As additional analyses χ^2^ tests for two-by-three tables with the row variable being “region” and the column variable being “project year” (baseline, first year after start of intervention, second year after start of intervention) were performed in order to evaluate effects of the awareness campaign on the outcome variables. These analyses are presented in several tables (S4, S5 and S6 Tables).

We used SPSS for Windows (version 20.0) for statistical analyses.

The significance level was set at α = 0.10 (two-sided). This significance level was selected because it was essential not to miss relevant effects in view of a low rate of suicidal acts associated with a high risk that clinically important effects are overlooked. We did not adjust for multiple comparisons when reporting p values for the regional interventions because we wanted to test specific hypotheses for each country (e.g., Leipzig versus Magdeburg).

**Ethical review.** The OSPI-Europe research project was executed in accordance with the principles laid down in the Helsinki declaration (2000). Each of the four research teams in Germany, Hungary, Ireland and Portugal sought ethical review and gained approval from the relevant bodies in each country: the Ethics Commission of the Medical Faculty, University of Leipzig, Germany (refs. 248–2007 and 140-2009-06072009); Semmelweis University Regional and Institutional Committee of Science and Research Ethics, Hungary (ref. TUKEB 149/2009), Ethics Research Committee of the Mid-West Regional Hospital, Limerick City and County, Ireland (no reference number, letter of approval dated 25/06/2009) and Clinical Research Ethics Committee, Merlin Park University Hospital, Galway City and County, Ireland (ref. C.A. 271); and the Ethical Committee of the Faculty of Medical Sciences, New University of Lisbon, Portugal (ref. CE/DP/7-2009). For the assessment of suicide attempts through patient records (Hungary, Portugal, partly Germany) or a routine procedure (Ireland) neither written nor verbal consent of patients was obtained. In case of interview participation, written informed consent was not obtained in order to not overwhelm the patients after suicide attempt with information and documentation. Informed verbal consent was obtained at the beginning of the interview by trained staff. A completed interview protocol functioned as documentation of participant consent. The ethics committee of each of the participating intervention regions approved this procedure prior to initiating the study.

## Results

### General effect on suicidal acts

A χ^2^ test revealed that the OSPI-Europe regional interventions did not have a significant global effect in terms of reducing the aggregated number of suicidal acts (χ^2^ = 0.02; df = 1; p = 0.89; see Table 4): The total absolute number of suicidal acts prior to the intervention in the intervention regions (1,781) declined to a mean number of 1,708 for the two years after its onset (percentage change: -4.10%; sum of population in the four intervention regions (in the year 2008): 1,046,112); similarly, the total absolute number of suicidal acts prior to the intervention in the four control regions (1,283) decreased to a mean number of 1,239 for the two years after the onset of the intervention (percentage change: -3.43%; sum of population in the four control regions (in the year 2008): 803,078). The same was true if the variable “region” was used as stratification variable (Cochran-Mantel-Haenszel test: χ^2^ = 0.02; df = 1; p = 0.90). Thus, the corresponding hypothesis was not confirmed (see also S4 Table). Furthermore, when looking at attempted suicides and completed suicides independently, no global effect was found for combined data from all four intervention regions (see Tables 5 and 6 as well as S5 and S6 Tables). The same was true for those attempted suicides using more lethal methods (χ^2^ = 0.36; df = 1; p = 0.55; see Table 5).

**Table 4:** Number of suicidal acts stratified for time period, region and country.

Region	Baseline	Means for the two years after onset of the intervention (SD)	p^a^
**All four countries**			
- Intervention region	1,781	1,708 (190.92) (-4.10%)	0.89
- Control region	1,283	1,239 (117.38) (-3.43%)	
**Germany**			
- Intervention region	491	465 (0.71) (-5.30%)	0.25
- Control region	180	196 (45.25) (+8.89%)	
**Hungary**			
- Intervention region	280	242 (23.33) (-13.57%)	0.75
- Control region	204	184 (25.46) (-9.80%)	
**Ireland**			
- Intervention region	737	767 (151.32) (+4.07%)	0.06
- Control region	677	612 (39.60) (-9.60%)	
**Portugal**			
- Intervention region	273	235 (16.97) (-13.92%)	0.05
- Control region	222	247 (7.07) (+11.26%)	

p, p value; SD, standard deviation. Data after adjustment for changes of gender-specific population figures in the intervention regions have been presented. Percentages are related to changes of the baseline values.

^a^ The p values (two-tailed testing) refer to the results of χ^2^ tests for two-by-two tables, with the row variable being “region” and the column variable being “time” (1 = baseline; 2 = arithmetic means for the two years after onset of the intervention programme).

**Table 5:** Number of attempted suicides stratified for time period, region and country.

Region	Baseline	Means for the two years after onset of the intervention (SD)	OR (95% CI) (p^a^)
**Attempted suicides in total**			
**All four countries**			
- Intervention region	1,643	1,545 (178.19) (-5.96%)	1.00 (0.90–1.11) (0.94)
- Control region	1,195	1,128 (112.43) (-5.61%)	
**Germany**			
- Intervention region	418	395 (7.07) (-5.50%)	0.82 (0.63–1.06) (0.12)
- Control region	155	179 (41.72) (+15.48%)	
**Hungary**			
- Intervention region	230	196 (14.14) (-14.78%)	1.03 (0.77–1.38) (0.85)
- Control region	169	140 (26.16) (-17.16%)	
**Ireland**			
- Intervention region	733	735 (146.37) (+0.27%)	1.16 (1.00–1.35) (0.05)
- Control region	669	577 (33.94) (-13.75%)	
**Portugal**			
- Intervention region	262	220 (24.75) (-16.03%)	0.73 (0.56–0.94) (0.02)
- Control region	202	233 (10.61) (+15.35%)	
**Attempted suicides with highly lethal methods**^**b**^			
**All four countries**			
- Intervention region	346	334 (47.38) (-3.47%)	1.08 (0.84–1.40) (0.55)
- Control region	185	165 (4.24) (-10.81%)	

CI, confidence interval; OR, odds ratio (control region/intervention region); p, p value; SD, standard deviation. Data after adjustment for changes of gender-specific population figures in the intervention regions have been presented. Percentages are related to changes of the baseline values.

^a^ The p values (two-tailed testing) refer to the results of χ^2^ tests for two-by-two tables, with the row variable being “region” and the column variable being “time” (1 = baseline; 2 = arithmetic means for the two years after onset of the intervention programme).

^b^ implying the exclusion of the lower-risk suicide methods “intentional drug overdose” and “use of sharp objects”

Significant country differences were found concerning intervention effects on suicidal acts (Breslow-Day test: χ^2^ = 8.83; df = 3; p = 0.03), due to heterogeneity of the corresponding odds ratios (OR) (Germany: OR = 0.87; 95% confidence interval (CI): 0.69–1.10; p = 0.25; Hungary: OR = 0.96; 95% CI: 0.74–1.25; p = 0.75; Ireland: OR = 1.15; 95% CI: 0.99–1.34; p = 0.06; Portugal: OR = 0.77; 95% CI: 0.60–1.00; p = 0.05).

**Table 6:** Number of completed suicides stratified for time period and region(for all four countries).

Region	Baseline	Means for the two years after onset of the intervention (SD)	OR (95% CI) (p^a^)
- Intervention region	138	163 (12.73) (+18.12%)	0.93 (0.65–1.33) (0.68)
)- Control region	88	112 (4.24) (+27.27%)	

CI, confidence interval; OR, odds ratio (control region/intervention region); p, p value; SD, standard deviation. Data after adjustment for changes of gender-specific population figures in the intervention regions have been presented. Percentages are related to changes of the baseline values.

^a^ The p values (two-tailed testing) refer to the results of χ^2^ tests for two-by-two tables, with the row variable being “region” and the column variable being “time” (1 = baseline; 2 = arithmetic means for the two years after onset of the intervention programme).

### Country specific intervention effects

Country specific intervention effects are shown for each of the four countries for suicidal acts (primary outcome) (Table 4) and for attempted suicides (secondary outcome) in Table 5. A significant effect in the expected direction of suicidal acts was found in Portugal. In Germany, a numerically relevant but statistically non-significant effect on suicidal acts in the expected direction was observed. For attempted suicides, a significant effect was again observed in Portugal: Whereas the number of attempted suicides decreased by 16% in the intervention region, it increased in the control region by 15%. In Ireland, a numerically relevant effect for suicidal acts was seen, with the rate increasing in the intervention area. The same was true for attempted suicides.

### OSPI-Europe theory of change

The OSPI-Europe intervention and its proposed theory of change (CIMO configuration) derived from the process evaluation is summarised below. Further detail is provided in S7 Table.

The overarching theory of OSPI-Europe is that single level interventions yield small impacts but combined multi-level interventions, or programmes, will yield greater benefits than individual interventions alone or may even achieve impact that is greater than the sum of their parts. Therefore, there is an implicit assumption of synergistic interaction between intervention levels, which found evidence for and reported elsewhere [16]. Additionally, the anticipated mechanisms of action are that the implementation of the OSPI-Europe intervention programme is facilitated by the simultaneous public mental health awareness campaign (to improve mental health literacy) and the development of local collaborative networks with individuals or organisations that have shared interests in the common goals of OSPI-Europe (that is, the reduction of suicidal behavior). These collaborative networks can facilitate access to target populations for the interventions and they will ‘buy into’, and therefore actively help, in delivering the OSPI-Europe interventions since they share the same goals of suicide prevention. The programme theory or anticipated mechanisms of action of the individual interventions differ, although in general, providing training to professionals in awareness of depression and suicidal behavior may increase levels of detection, referral and support (Levels 1 and 3); while the mental health literacy campaign (Level 2) seeks to transform knowledge and awareness of mental health issues with the intent of reducing stigma associated with a mental health diagnosis. The mental health literacy campaign may also encourage help seeking and community support for those with mental health issues, complementing the Level 4 activity that seeks to change the behaviour of those at risk, and to develop locally driven action plans for restricting access to means of suicide.

### Implementation of the 4-Level approach across case study sites

Table 7 summarises the key findings for each study site in relation to the core functions of the process evaluation (CIMO) and for each of the levels of intervention activity within OSPI-Europe.

**Table 7:** Observed context, implementation, mechanisms (of implementation) and outcomes (CIMO) by level of intervention.

	Context	Implementation	Mechanisms	Secondary outcomes	Primary outcome
**Overall**	General recession across Europe but across country differences (see also below): e.g in Ireland a large company closure in the intervention region may have escalated mental health issues in those recently unemployed and contributed to a lower impact of the interventions; Portugal’s recession meant a lack of resources for welfare payments increasing hardship and potential mental health problems in affected families. Across country differences in health and social care systems.	OSPI imple-mentation goals generally met/ exceeded.	OSPI-EUROPE leads are respected locally with degree of social capital. Local Advisory Groups, receptive or already aligned to OSPI-Europe goals, are established in most regions to support implementation. Synergistic interactions between levels leading to enhancement/ added value of individual level activity or ‘new’ activity beyond original programme plans.	Secondary level outcomes varied by level of intervention and by country but in general the GP and CF training (Levels 1 and 3) were effective in changing attitudes and confidence but raising public awareness was less decisive due to large country variations (see examples below).	OSPI-Europe interventions did not have a signifi-cant global effect in the reduction of the aggregated number of suicidal acts. Significant country differences were found concerning intervention effects on suicidal acts. A significant effect in the expected direction was found in Portugal for both suicidal acts and attempted suicides. A nonsignificant trend in the expected direction was found for Ger-many and Hunga-ry. For Ireland, a trend in the opposite direction was found.
**Level 1 GP training**	Different levels of engagement with GPs across regions. Engagement ‘hard work’ in regions in Ireland and Portugal. Similar GP training had already taken place in Ireland. Different baseline attitude scores between countries. GPs in Hungary had more negative perceptions of depression and its treatment and the lack of capacity in psychiatric services meant that although they had improved confidence in detection/diagnosis, there were no/little referral options.	Minimum intensity mostly met in Germany and exceeded in 3 regions. Training duration and type of trainer differed slightly across regions: delivered by psychiatrists in Germany and Portugal; GP peers in Hungary and psychologists in Ireland. Some TtT sessions occurred in Germany (and a small number in Portugal) to enable future sustainable delivery.	Academic respect of local OSPI-EUROPE leads and perceived model of training as ‘evidence based’. Adaptation of training delivery (e.g. reduced to 2 hour ‘refresher course’ in Ireland). Local (respected) GP champion in Hungary successfully engaged GPs. High level gatekeepers such as those leading CME helped with both access to GPs time and selling the need for further training.	Significant improvements were observed in attitudes towards depression and suicide prevention and confidence in dealing with suicidal individuals. At 3 months follow-up, GPs increased confidence to deal with depression and suicide was maintained whereas their attitudes towards depression and suicide prevention had returned to baseline. German GPs were most likely to maintain training effects. No GP data available for Ireland.	
**Level 2 Public awareness**	Intervening contextual factors (death of public figure in Germany and flooding and elections in Hungary) may have impacted on visibility of campaigns: increasing visibility and awareness in Germany and reducing visibility and awareness in Hungary. Similar national campaigns in Ireland likely to have impacted on control regions.	Minimum intensity slightly reduced in Germany and Ireland, met in Hungary and exceeded in Portugal. Fewer public engagement events than anticipated.	Support and engagement of other collaborative partners helped to disseminate materials, especially in Ireland. Active support and resources from the local council in Portugal. In Hungary, mere replication of campaign materials and blanket distribution across region may have failed to reach appropriate populations. High levels of media coverage of topic area across Germany. Local volunteers in Germany increased the impact of the campaign and added capacity to public events.	Campaign significantly visible in Germany and Portugal, visible but not significantly visible in Ireland, and not visible in Hungary. Hungary consistently demonstrated no improvement in terms of mental health literacy, with Germany consistently showing improvement. Overall, large country differences were observed, with some improvement also observed in control regions.	
**Level 3 Community facilitator training and TtT**	Local politics hampered inclusion of specific groups in some countries. Over half of the police officers in Ireland had been exposed to suicidal behavior, compared with just over 6% of those in Germany where the participants were police officers in training.	More than double minimum intensity achieved in all regions. In Hungary and Ireland the training was mostly peer-led whereas in Portugal and Germany no train-the-trainer sessions occurred (training was delivered directly by members of the OSPI-Europe team).	High affinity with topic of suicide among partner organisations. Partner organisations recognised needs within their organisation: especially staff on front-line for dealing with suicide crisis. E.g. Police was a key part of the community in 3 regions in terms of their exposure to the most vulnerable and distressed persons. Strong links with specific collaborators influenced recruitment of CFs and adoption of TtT.	Significant improvement in attitude, knowledge, and confidence and maintained at 3 months but more than half of CFs were from German intervention site. There were significant country and occupational differences which also varied across measures, e.g. Hungarians had least favourable attitudes towards depression but were most confident in dealing with suicide.	
**Level 4 interventions for patients, high-risk groups, and their relatives.**	Cooperation with other (acute) health sectors was necessary but not always evident or achievable.	Countries varied in interventions delivered but most relied on accessing existing local resources (helplines).	Patient and family peer support network acted as catalyst for activity in Germany which supported other intervention levels.	No secondary outcomes collected.	
**Locally based actions on restriction of access to means.**	Cooperation with other multiple organizations was necessary. Ireland’s highly inclusive model facilitated such partnerships.	Each intervention site had its own focus which required different types of intervention and different types of collaboration.	Actions at this level require high level ‘ownership’ to the extent they fund preventative actions as well as high level ‘authority’ to approve actions. Some stakeholders had been engaged (e.g. in identifying a local site where suicides had occurred) but ‘ownership’ and resourcing to tackle the problem had not followed and sufficient ‘authority’ had not been engaged. Ireland’s organizational model brought stakeholders together where collective ownership and authority was achieved (between the police and water authority) and led to preventative actions.	Actions at this level were not completed. Some emergency telephone numbers made visible at hotspots. No secondary outcomes collected.	

CF, community facilitators; CME, continuing medical education; GP, general practitioners; OSPI(-Europe), optimizing suicide prevention programs and their implementation in Europe; TtT, train-the-trainer seminars.

For Level 1 activity, the delivery of GP training to the intended numbers was achieved but not without significant effort in some regions (Ireland and Portugal). In Ireland the training required adaptation to a much reduced 2 hour ‘refresher’ training and also required the help of the local Continuing Medical Education (CME) coordinator to convince GPs this additional training was necessary. The ‘reputational capital’ of the OSPI leads, or the local champions they engaged to help with recruitment and delivery, also played a significant part in obtaining buy-in from GPs. In Germany and Hungary the training was perceived as ‘evidence based’ and trustworthy. Portugal relied on high level gatekeepers in Psychiatry and Primary Care reaching a collaborative agreement which then made participation in the training mandatory for GPs. This approach could have resulted in reluctant attendees, however, limited prior training in mental health in Portugal meant that the training was perceived as being of value at the end of the day.

*But actually one thing I noticed GPs were eager to have a real precise orientation on [case studies] of patients*. *They wanted to comment on cases and to have clear guidelines to choose drugs on some situations and not others*, *and I think they were really interested in having that kind of training*. (FG3-6).

Although Hungary recruited GPs more easily, they found that the training did not address the fundamental lack of capacity for referral options which left some GPs feeling powerless. This may explain the lack of sustained change in attitudes and confidence immediately post training.

*there are suggestions [that it might now be] even worse because now they know that there is a problem that they cannot solve*. (FG2-6).

The provision of training alone is not sufficient to ensure its uptake. It most often requires a local champion or high level gatekeeper to promote its validity and the professional status of the trainer was important in some countries (such as Portuguese GPs expecting and responding more to a Psychiatrist as trainer) but not all (in Ireland this was not highlighted as relevant).

As hypothesised, the use of collaborative partners was instrumental in increasing capacity and distribution within campaigns. In Portugal for example, the public campaign dissemination was helped to a large extent by good relationships with the local council who helped to produce and distribute campaign materials.

*Because we have framed the times and period and numbers of posters… and the way they were [distributed] we have all that registered*. *So I would say the council entered with many thousands of Euros in the campaign*. (FG3-6).

In Ireland, the OSPI-Europe campaign provided much needed information resources which organisations could then distribute on their behalf.

*‘Well we sent leaflets to schools*, *sports bodies*, *sporting organisations*, *libraries*, *health services*, *GPs*, *……the feedback we got was they were so delighted with it because now there’s not a lot of resources to produce resources like that*, *so a lot of organisations are really struggling to print even their own leaflets*.*’* (FG1-6).

Establishing good relationships with local media assisted take-up of reporting guidelines (also part of public awareness raising activities) also provided an avenue for free distribution of leaflets.

*I was surprised by the access to the media and the journalists*, *many of them were there at the [public event] launch …*‥*And then through them*, *…we were able to achieve that twice posters and leaflets were put for free*, *almost for free*, *maybe 200 Euros*, *thousands of copies in a newspaper and it was a door to door newspaper*. (FG3-6).

However, collaborating with organisational partners in distribution activities did not necessarily ensure ‘engaging’ with the general public: this was seen as requiring additional effort.

*I think we probably didn’t do enough public events*, *I would’ve liked to have done more but*, *you know*, *time*, *it’s actually quite difficult to organise proper events*, *they require a lot of planning and a lot of organisation*. *So I’d liked to have been able to do a little bit more of that*. (FG1-6).

It is in relation to Level 2 activity (public awareness) that the issue of ‘context’ has proved most pertinent. In Germany, a key intervening contextual event was reported during the intervention period: the death of Robert Enke, the German national goalkeeper. This raised awareness of suicide and depression nationally and the coverage was considered to have likely reduced stigmatising attitudes over the country as a whole. The impact of this event has been verified in data on subsequent railway suicides and in a separate analysis of media reporting of suicide [51–55]. In a team meeting, the footballer’s death was discussed at length.

*Many effects in the [public] attitude scales were found in the intervention region as well as the control region*. *[Researcher’s name] noted that in Germany this may be due to the suicide of Robert Enke as after this there were a lot of articles about depression in the media reported nationally over a period of months (Fieldnotes*, *Meeting 14/11/2011)*.

Participants in Ireland also reported an intervening event or action which may have reduced the visibility of the OSPI-Europe public awareness campaign: the existence of a simultaneous mental health awareness campaign being run nationally (Your Mental Health). The intervention region in Ireland was also highly affected by the economic recession occurring at this time with the closure of its largest employer [56]. Many of the key stakeholder partners in the intervention region voiced their concerns about the impact of the recession on suicide.

How equipped are 30/33 year olds now to actually deal with more difficult times? And if, in a period of boom, you have plenty external stimulants on which you could actually hang your own self-esteem and value for yourself in terms of- the type of car I drive—when they're gone what are you left with? And so you're back into probably some of the underlying vulnerabilities that might shape a person who is at risk of self-harm and suicide in terms of hopelessness and things like that.(I: Int1-1)

Overall these (unanticipated) local contextual events are likely to impact on observed outcomes in both intervention and control regions.

In Hungary, baseline levels of mental health literacy were significantly lower than other sites [28]. Additionally, two major local events may have reduced visibility of the campaign. These were, a major flood in the intervention region and a significant local election with associated (poster) campaigning which drew public and media attention away from the OSPI-Europe campaign.

*The public campaign was not as intensive as they would have liked as it happened at the same time as the general election, when their materials were swamped by electioneering materials (Fieldnotes, Meeting 10/5/2010)*.

Therefore, even when the hypothesised ‘mechanisms of action for implementation’ are achieved, this may not necessarily transfer to recognition and engagement by the public, and intervening contextual events may easily override campaign plans for better or for worse.

Level 3 activity Community Facilitators (CF) training was also successfully delivered in each intervention region at the planned levels. One major difference between countries was in the type of CF trained: with police representing the majority of those trained in Ireland and Portugal but only a minority in Germany and Hungary respectively. Indeed, the reported local events in Hungary (the flooding and local elections) were specifically named as limiting police availability for training.

As hypothesised, Ireland implemented the train-the-trainer model in a manner that ensured that the local groups took ownership of OSPI. Sustainability of the training intervention was achieved by training key gatekeepers who could then roll this out, therefore also generating capacity for suicide prevention.

*‘we trained only five senior [staff] in the Limerick police and those five police officers trained close to 500 of their own peers’*. *(OSPI Optimising Workshop Presentation 2)*.

While delivering the core components of the training, the team then also gave time to delivering material specifically tailored to the needs of the particular professional groups. This enhanced relevance, take up and may help to maintain sustainability of the programme.

*‘we make sure that we achieve all the basic information that we provide in the training*, *but after the break we are able to discuss also issues that are specific to these groups*, *and I think again in the police force that was a very good example’*. (OSPI Optimising Workshop Presentation 2).

The implementation of CF training was clearly influenced by the level of buy-in from specific stakeholder organisations and different countries nurtured different sets of relationships. The transformation of some gatekeepers into stakeholders with ‘ownership’ of OSPI-Europe activities was most pronounced within the uptake of CF training and this transformation was particularly successful in Ireland where the ‘Train the Trainer’ (TtT) model became peer-led, therefore making it highly sustainable beyond the life of OSPI-Europe. However, in implementing the CF training across all sites it was acknowledged that there were different training needs among CF’s who had different levels of baseline knowledge (knowledge needs) which needed to be taken into account when designing and delivering training.

Level 4 activity on interventions for at-risk populations and local action to restrict access to means differed for each region as there was no ‘common’ intervention beyond guidance for the use of ‘emergency cards’ distributed via hospitals, and the use of (mainly existing) help-lines. Quantification of these activities was difficult as they often resided within the control of other organisations (e.g. hospitals distributing cards). However, there were some unique developments within individual sites: for example, existing peer-led self-help groups in Germany were expanded through OSPI-Europe participation and support, and participants helped to create synergistic interactions between Level 4 and Levels 1, 2 and 3 activity [16];

*They [self-help groups] are a big help for us—in our plan we intended to help them, but it turned out they have helped more us than the other way round (G: Int 1–3)*.

In Ireland, collective action/interaction on restriction of access to means by the police and the Water Authority was facilitated by the local collaborative network established by OSPI-Europe to galvanise action to reduce suicides occurring in the local river.

Local activity around restriction of access to means was found to be difficult to achieve as this ultimately required both substantial financial resources and influence at a strategic or policy level. Securing the commitment to such resources was beyond the scope and timescale of the OSPI-Europe intervention. However, the topic of access to means was often raised with professional groups in Level 1 and Level 3 activity.

The OSPI-Europe theory of change had hypothesised that synergistic effects might be observed between intervention levels and this was evident in the enhancements that patient and public involvement (at level 4) brought to Level 1, 2 and 3 activities [57]. Conversely, one-off or time limited implementation of some interventions such as public campaigns will be unlikely to result in sustained change, especially when there is potential for unanticipated intervening contextual events to impede the impact of interventions.

## Discussion

Our main hypothesis was not confirmed: when aggregating suicidal acts from all four intervention regions, suicidal acts did not show a statistically significant reduction compared to a one-year baseline and the control regions. However, intervention effects differed significantly between countries. At country level, a statistically non-significant effect on suicidal acts was found in Germany (intervention region: - 26; control region: + 16) and a numerically and statistically significant effect in Portugal (intervention region: - 38, control region: + 25). In Hungary, although rates decreased in both intervention and control area, no specific positive effect of the intervention was seen and even a significant effect in the opposite direction was seen in Ireland (intervention region: + 30, control region: - 65).

Possible reasons for the differences in outcome between the OSPI-Europe study and previous studies suggesting significant positive effects of a multilevel suicide prevention program on the frequency of suicidal acts, especially the study of the effects of the Nuremberg alliance against depression [20] refer to the intensity of the intervention (e.g., about 2000 trained community facilitators in Nuremberg versus 915 in Leipzig, both with about 500.000 inhabitants) and major intervening factors identified in the context of the OSPI project (e.g., flooding and elections in Hungary). Comparable intervening factors had not been noticed in Nuremberg. However, a process analysis comparable to that in the OSPI project had not been implemented in Nuremberg and it cannot be excluded that such factors played a relevant role in Nuremberg. Moreover, according to the results of a Breslow-Day test, the intervention effects in Nuremberg as compared to the control region (OR = 0.759; 95% CI: 0.600–0.960) did not significantly differ from the corresponding effects in Leipzig as compared to the control area of Magdeburg (OR = 0.870; 95% CI: 0.685–1.104) (χ^2^ = 0.634; p = 0.426). Thus, this post-hoc explanation has to be regarded with caution.

The significant country differences concerning intervention effects point to the relevance of contextual factors as well as differences in the implementation process. Several external and mostly unforeseeable events which likely interfered with the OSPI-Europe interventions were identified by the OSPI-Europe process analysis:

-  In Germany, the national football goalkeeper Robert Enke, who had a depressive disorder, died by railway suicide in November 2009. His death was followed by extensive media coverage, including the nationwide broadcasting of the public funeral, which was held in a large football stadium. This resulted in copycat effects [52,53], with a long-term increase of railway suicides (from 2.30 per day in the two years before Enke´s suicide to 2.73 per day in the two years after) [52]. The suicide occurred in between the two general population surveys performed within OSPI-Europe, so that the effects of media reporting on knowledge and attitudes in the general population could be estimated. As reported by Kohls et al. [29], the personal stigma decreased considerably and the value of professional help increased remarkably in Germany—in both, the intervention and control region [29], allowing the assumption that the long-lasting, intensive and in many cases sensible and informative media coverage after the suicide of Robert Enke functioned as a large overall depression awareness campaign which overshadowed possible effects of the local activities in Leipzig.
-  In Hungary, a serious flood in spring 2010 in the intervention region during the first intervention year, as well as national elections for parliament in April and for local government in October 2010, were identified as events that strongly interfered with the intervention. The flood and the elections dominated media reporting and made it difficult for the public awareness campaign in the intervention region to get noticed. The OSPI-Europe general population survey revealed that in the intervention region in Hungary only 8.6% of the people had noticed the intervention activities, a number which is lower than that observed in the other intervention regions (Germany: 25.8%, Portugal: 23.6%, Ireland: 11.2% [29]). The process evaluation furthermore revealed that these events also impeded other intervention activities, e.g. very few police officers (n = 13) participated in community facilitator training due to the need to respond to the flood and its aftermath. This compared with 134 police officers trained in Germany, 494 in Ireland and 302 trained in Portugal. Furthermore, there were no significant changes in negative attitudes toward depression and mental health stigma in the Hungarian intervention and control regions [29].
-  In Ireland, respondents in the process evaluation reported that the impact of the economic recession and subsequent austerity may have had a particularly negative impact on people residing in the intervention region as the region’s single largest employer closed down in 2009. As a result, the intervention region became an unemployment ‘blackspot’ [57], and in 2011 it had the highest unemployment rate in the country (29%) [56]. This may have had consequences concerning access to care for people with depression and other mental disorders. In Ireland it was also difficult to isolate the impact of OSPI-Europe as it was implemented alongside a national suicide prevention campaign, which made it difficult to achieve differential effects between the intervention and control sites.
-  In Portugal, the economic crisis hit both the intervention and the control region.

One relevant factor explaining the fact that an intervention effect was noted for Portugal but not for any other sites could be that the percentage of individuals having self-reported experience with depression, deliberate self-harm and suicides in relatives was the highest (66%) in Portugal in comparison to Germany, Hungary and Ireland [29]. Thus, the public alertness to the target messages on the depression-related campaign seems to have been highest in Portugal and to have contributed to a significant effect of the multi-level intervention program regarding suicidal acts.

Another important factor has to be considered when discussing findings on suicidal behaviour obtained in such complex community based interventions. Intervention measures such as the training of professionals and the public awareness campaign can induce a bias by improving the recognition of suicidal acts in the intervention regions. Whether or not a certain behaviour (e.g. on a bridge) or medical condition (e.g. intoxication) is documented as an attempted suicide is not independent of the awareness and knowledge of involved people such as GPs or policemen. This could result in an artificial increase in the rate of suicidal behaviour. The Nuremberg Alliance Against Depression results had pointed to such a bias: the intervention effects were smaller for suicidal behaviours involving drug overdose than for more lethal suicidal methods. It is possible that more intoxications were recognized as suicidal behaviour in the intervention region due to the combination of the professional training and the public mental health campaign. In the case of more lethal means of suicide, this bias is expected to be lower because such suicidal behaviour is more easily recognized [20]. In the present study, sub-analyses of aggregated data over all four countries did not reveal more pronounced intervention effects for more lethal suicide methods. For Ireland, however, a recognition bias offers a possible explanation for the finding of increased suicidal acts in the intervention compared to the control region. This bias might have been large in Ireland because 66.10% of policemen and nearly all general practitioners (GP) participated in the trainings whereas the corresponding rate in Germany was clearly lower (10.36% of policemen, 10.45% of GP) [30]. The possibility exists that an increased recognition of attempted or completed suicides by policemen and GPs has inflated the statistics in the intervention region compared to the control region in Ireland.

The results should be interpreted alongside the secondary outcomes results: The OSPI-Europe training program led to improved attitudes towards and treatment of depression and suicide prevention and enhanced confidence in GPs engaging clinically with depression and suicidality [27]. Kohls et al. [29] revealed that the public depression awareness campaign had been noticed by the general public and that respondents in the intervention regions were characterized by significantly lower personal depression stigma than respondents in the control regions after onset of the OSPI interventions. Moreover, Harris et al. [16] demonstrated that all four countries which had implemented the OSPI-Europe project achieved synergistic impacts that added value beyond the sum of single intervention levels or isolated components. Thus, it is unlikely that the OSPI-Europe interventions lacked effectiveness; instead we have to conclude (see [58], page 179) that “the complexity of the synergistic causal chains in multi-level community-based interventions makes it rather unfeasible to single out the specific size of the contribution to the suicide preventive effect of a certain measure in the entirety of the multi-level intervention” as having aimed by Krysinska et al. [59]. An in-depth economic evaluation of multi-level suicide prevention activities would allow the assessment whether these activities were closely linked to reductions in attempted and completed suicides [60]; such an analysis was planned and could supplement our findings in a significant way.

Negative results of an evaluation of multi-level interventions for suicide prevention in New Zealand (MISP-NZ) were reported recently [24]. Drawing comparisons between this and the OSPI-Europe study is difficult due to marked differences in study design. Whereas the OSPI Intervention combined two partially overlapping aims, namely improving care for people with depression and preventing suicidal behavior, the MISP-NZ study focused only on suicide prevention. One advantage of combining both aims is the possibility to focus more on depression, when targeting the general public and equally to depression and suicide when targeting community facilitators and GPs. There is still uncertainty what is balance between positive and negative effects of public relation campaigns focusing in suicide prevention. Even when care is taken to avoid negative reporting in line with recommendations of media guides [1] the spreading of this theme in social media cannot be controlled and might lower the threshold for suicidal acts. Furthermore, several crucial elements such as creating an ownership feeling in the intervention region by e.g. an opening ceremony or strong engagement of general practitioners were lacking within the MISP-NZ activities.

### Strengths and limitations

OSPI-Europe represents one of the first international studies to address the methodological complexity of multifaceted community based suicide prevention interventions by implementing a multi-level evaluation design along with a multi-level intervention model. Systematic process and context evaluation together with the assessment of a series of secondary outcomes allows a more nuanced discussion of the primary outcome findings. The international approach as well as the efforts to standardise the interventions in advance of implementation are further strengths of this study.

There are the following limitations to report:

1. The low statistical power due to the population size in the intervention and control regions and the low base rate for suicidal behaviour result in the risk that relevant clinical effects have been overlooked.
2. Suicidal acts diagnosed and treated outside of hospital settings were not assessed due to practical reasons. Consequently, there was not a complete assessment of attempted suicides. However, this problem is limited by the fact that changes over time rather than absolute numbers of suicidal acts were considered and care was taken to keep the assessment of attempted suicides stable over time.
3. The intervention and respective control regions were different in size and sociodemographic structure as well as regional context. Since changes over time rather than baseline differences were considered in the primary outcome evaluation, the risk of bias introduced by these differences is limited but cannot be definitively excluded.
4. A stratification of analyses by a financial measure and baseline differences in terms of several socio-demographic variables like age, gender, socio-economic status, and civil status would have been of interest but was not feasible due to the limited absolute numbers of suicidal acts in the selected intervention and control regions.
5. The duration of the intervention period (1.5 years), which was significantly shorter compared to the initial implementation of the 4-level suicide prevention programme in Nuremberg, might not have been sufficient for some intervention effects to become visible.
6. Although activities related to limiting access to suicide ‘hot spots’ were initiated during the project, the impacts of these are slow to realise given the negotiations with local authorities and structural changes required (e.g securing a bridge against suicide attempts). Therefore the potential impact of these activities were unable to be captured during the evaluation period.
7. A further methodological limitation is related to the fact that data on the incidence of relevant mental disorders like major depression or borderline personality disorder had not been gathered in the cities of interest; thus, we could not answer the question whether differences between countries regarding the efficacy of the OSPI-Europe intervention program could be partly explained by regional differences in the incidence of mental disorders known to be closely associated with suicidal acts.
8. It would have been interesting to look for events during the baseline periods that could have impacted local suicide rates in a systematic manner. This could be an important aspect for the design of future studies regarding the evaluation of a multi-level program for suicide prevention.
9. The discussion of possible intervening factors identified by the process analysis and context evaluation is done post-hoc, partly based on qualitative data only and therefore associated with uncertainty.
10. The local OSPI teams also considered access to lethal means by identifying suicide ‘hotspots’ and worked with local authorities to support suicide prevention measures. However, this often involved changes to infrastructure (like barriers on bridges) that involved time consuming burocratic decision processes which could not be finished within the time frame of OSPI-Europe.

### Conclusions

When aggregating data across countries, no significant effect of the OSPI-Europe multi-level suicide prevention programme on suicidal behavior was found. Therefore, the first hypothesis was not confirmed. At the country level, the previously observed preventive effects on suicidal behaviour of the 4-level intervention concept were replicated but only in one of the four intervention regions. Contextual factors, independent process evaluation and secondary outcomes have to be taken into account when interpreting findings of such a complex community based intervention. They deliver plausible albeit only post-hoc explanations for the country differences concerning the preventive effects of the intervention on suicidal behavior.

A lesson learned by OSPI-Europe is that findings from complex community based multi-level interventions do not allow a straightforward interpretation of cause and effect, but require in-depth interpretation in the light of secondary and process analysis data which take account of the context in which these complex interventions are delivered. Such post-hoc analyses cannot provide evidence at the same scientific level as randomised controlled drug trials but they are crucial in order to avoid misleading interpretations which might result when considering the main outcomes in isolation and without taking regional and national circumstances into account.

## Supporting information

S1 Checklist STROBE statement—checklist of items that should be included in reports of observational studies. (RTF)

S1 Table Changes of population in the OSPI-Europe intervention and control regions (2008–2011). (RTF)

S2 Table Differences between OSPI-Europe intervention and control regions regarding changes of the corresponding populations between 2008 and 2011. (RTF)

S3 Table Number of suicidal acts after adjusting for changes of gender-specific population figures in the intervention regions. (RTF)

S4 Table Number of suicidal acts stratified for project year, region and country. (RTF)

S5 Table Number of attempted suicides stratified for project year, region and country. (RTF)

S6 Table Number of completed suicides stratified for project year and region. (RTF)

S7 Table OSPI-Europe theory of change. (RTF)

S8 Table Core and optional intervention measures of OSPI-Europe. (RTF)
