# Supplementary material for: Prevention of suicidal behaviour: Results of a controlled community-based intervention study in four European countries
Source: PLoS One. 2019 Nov 11;14(11):e0224602. doi: 10.1371/journal.pone.0224602 (PMC6844461; doi:10.1371/journal.pone.0224602)
Supplement: S1 Checklist — (RTF) [file pone.0224602.s001.rtf]

S1 Checklist. STROBE Statement—checklist of items that should be included in reports of observational studies.
	Item No.	Recommendation	Page 
No.	Relevant text from manuscript	
Title and abstract	1	(a) Indicate the study's design with a commonly used term in the title or the abstract	1	See title: “controlled community based intervention study in four European countries”	
		(b) Provide in the abstract an informative and balanced summary of what was done and what was found		3-4	“The community-based intervention concept of the 'European Alliance Against Depression' simultaneously targeting depression and suicidal behaviour has 4 levels: training of general practitioners, a public awareness campaign, training of community facilitators, and support for patients and their relatives. Two main aims of the project “Optimizing Suicide Prevention Programmes and Their Implementation in Europe” (OSPI-Europe) funded by the European Union were to optimise this approach and to evaluate its efficiency and contextual determinants. The aim of this paper is to present the primary outcome results of this project: effects of the intervention on the number of suicidal acts (completed and attempted suicides combined). 
Interventions were implemented in four European cities (Leipzig, Miskolc, Amadora, Limerick). Changes in frequency of suicidal acts with respect to a one-year baseline were compared to four control regions (chi-square tests). In addition, the comprehensive evaluation model comprised primary and secondary outcomes as well as an independent process evaluation. 
The overall decrease in suicidal acts compared to baseline in the intervention regions (-58 cases, -3.26%) did not differ significantly (2 = 0.13; p = 0.72) from the one in the control regions (-18 cases, -1.40%). However, intervention effects differed between countries (2 = 8.59; p = 0.035), with significant effects on suicidal acts in Portugal (2 = 4.82; p = 0.03). 
The findings illustrate the problems inherent to evaluating complex community based interventions. The fact that the intervention programme failed to reach an overall significant reduction of suicidal acts, might be due to uncontrolled intervening factors such as national elections and flooding in the intervention region in Hungary, as well as a celebrity suicide in Germany as identified during the accompanying process evaluation. Nevertheless, some of the results at country level are promising.” 	
Introduction		
Background/rationale	2	Explain the scientific background and rationale for the investigation being reported	5-8	“Completed and attempted suicides remain to be a significant mental and public health issue. In 2015, close to 800,000 people worldwide died by suicide, 58,000 of them in Europe [1]. The number of attempted suicides is estimated to be more than 20 times higher [1]. Suicidal behaviour occurs mostly in the context of mental illness, with depressive disorders being most prevalent [1,2]. At this point, around 30 million European citizens suffer from unipolar depression per year [3], most of them with no or suboptimal treatment. To improve the care for people with depression is therefore a central element in suicide prevention strategies [4]. 
The intervention strategy applied within the research project OSPI-Europe (“Optimising Suicide Prevention programmes and their Implementation in Europe” funded by the European Union (EU), 7th Framework Programme) is based on the experiences and results from a model project in Nuremberg. During an intense 2-years 4-level community-based intervention in the city of Nuremberg (480,000 inhabitants), a significant reduction of suicidal acts (- 24 %, primary outcome) was found in Nuremberg compared to both a control region (Wuerzburg, 270,000 inhabitants) and a baseline year. This effect turned out to be sustainable [5,6]. Following these positive results, such community based 4-level interventions have been implemented in more than 100 regions in Germany and other countries in and outside of Europe (www.eaad.net) (reviewed in [7]). Further evidence for the suicide prevention effect of this approach has been provided from other regions in Germany and Hungary [8,9]. 
The OSPI-Europe intervention [10] was implemented in four cities (Leipzig, Miskolc, Amadora, Limerick) in Germany, Hungary, Portugal and Ireland respectively. It comprised actions at four levels: 1) primary care providers, 2) the general public via public awareness campaigns, 3) community facilitators and gatekeepers to secondary care, and 4) patients, their relatives and high-risk groups such as survivors of a suicide attempt. Consistent with a systematic review conducted within OSPI-Europe [11], “restricting access to lethal means” was another element included in the programme.
Multi-level interventions can also be considered 'complex interventions'. Not only do they involve implementing various actions simultaneously, but they also require a multi-level evaluation approach. For several reasons this methodological approach is different from that used in a conventional randomized controlled trial: 
-	Unforeseen regional and national circumstances or events can interfere, facilitate or impede both the implementation process and the effectiveness of the intervention (see S1 File). 
-	The different components of the intervention may act independently or synergistically, complicating identification of the ‚active component(s)' [12,13]. Unanticipated effects go beyond the pre-determined intervention plan and catalytic effects may arise. For example, health political decision processes might be facilitated or new self-help activities might be triggered, leading to unplanned additional intervention elements [13]. 
-	The intrinsic motivation and enthusiasm of those running the regional intervention is likely to be an important element in a successful intervention. These elements are at risk to get lost when interventions occur within a randomized controlled design.
-	OSPI-Europe tried systematically to take this into account and to assess the complex social world where these interventions are implemented [14,15]. Therefore, a systematic process evaluation was conducted in OSPI-Europe. Contextual information together with secondary and intermediate outcomes can provide insights into their relative effectiveness and help to understand the implementation process itself and how the different intervention elements work together. Results concerning intermediate outcomes such as public attitudes toward depression and help-seeking, as well as some initial results from the process evaluation, have already been published [12,16-20]. In this issue the results concerning primary outcome will be published together with those of the main process evaluation results. The latter are of importance for the interpretation of the results concerning primary outcomes and contribute to understanding the methodological complexity of conducting process and context evaluation of multilevel interventions. 
In this article the focus will be on the primary outcome. The combination of attempted and completed suicides (suicidal acts) was chosen as primary outcome because the base rates for completed suicides alone is low, resulting in low statistical power and the risk to miss clinically relevant effects. The following hypotheses were tested: 
Compared to the baseline year, the two-year 4-level interventions result in a reduction of suicidal acts (completed + attempted suicides) which are stronger than corresponding changes over the same time period in control regions. This intervention effect was tested for data aggregated for all four intervention and four control regions as well as independently for the four intervention and the four corresponding control regions. 
Aim of the EU-funded OSPI-Europe project (carried out between 2008 and 2013) (for details see [10]) was to strengthen the evidence that the 4-level interventions reduce suicidal behaviour, but also to gain a better understanding of the determinants for successful implementation and of the effective components of the 4-level intervention in different cultures and healthcare systems. This aim had been achieved.”	
Objectives	3	State specific objectives, including any prespecified hypotheses	8	“The following hypotheses were tested: 
Compared to the baseline year, the two-year 4-level interventions result in a reduction of suicidal acts (completed + attempted suicides) which are stronger than corresponding changes over the same time period in control regions. This intervention effect was tested for data aggregated for all four intervention and four control regions as well as independently for the four intervention and the four corresponding control regions. 
Aim of the EU-funded OSPI-Europe project (carried out between 2008 and 2013) (for details see [10]) was to strengthen the evidence that the 4-level interventions reduce suicidal behaviour, but also to gain a better understanding of the determinants for successful implementation and of the effective components of the 4-level intervention in different cultures and healthcare systems. This aim had been achieved.” 	
Methods		
Study design	4	Present key elements of study design early in the paper	13-14	“In each of the four selected European countries in this analysis (Ireland, Portugal, Germany and Hungary) an intervention and control region was chosen. Selection was based on population size (having at least 150,000 inhabitants in each geographical area; see Table 2) and the presence of regional partners interested in cooperating within OSPI-Europe. Selection of control regions was guided by the aim to match the intervention regions concerning population size. Additional requirements were that no previous suicide prevention or depression awareness programme had taken place and that the local stakeholders/researchers were interested in participating in the project.”	
Setting	5	Describe the setting, locations, and relevant dates, including periods of recruitment, exposure, follow-up, and data collection	8-15	“The OSPI-Europe intervention
The study compared a one-year baseline period to a two-year index time period after start of the intervention. Comparisons were made with both the baseline period and with the control region. Intervention initiation was marked by a public opening ceremony for each intervention region. Intervention activities were conducted for a minimum period of 1.5 years.  
A list of required and optional interventions was defined (see S1 Table). Table 1 shows the intensity of mandatory activities for intervention levels 1 to 3. Several optional actions were undertaken depending on local needs and circumstances. For level 4, activities such as supporting self-help groups were carried out. Efforts to restrict access to lethal means included the local identification and security inspection of locations where people frequently take their lives.  
[…]
The public awareness campaign addressed primarily depression by focusing on the messages: “Depression is a real disease.”; “Depression can affect anyone.”; “Depression has many faces.” and “Depression can be treated.” [19]. The campaign comprised an opening ceremony, public events with the aim to inform about depression, distribution of flyers and posters and a close cooperation with the local press [19]. Running of the same campaign in different regions was not possible due to cultural and financial differences leading to different intensities of the campaign in the four intervention regions.
Regarding the training of general practitioners and community facilitators, the duration of the training was 120-240 minutes. It had the aim to inform about depression and suicidal behavior, to explain the difference between mental health and mental health suffering, to perform interventions in the case of a crisis, and to educate bereaved relatives, with role-plays being a relevant part of the training. The trainings in the four intervention regions had been adapted to the local health care system (for details see [18]).
Regarding interventions for patients, relatives and survivors of suicide attempts, they mainly consisted of providing of an emergency card and advancement of both self-help groups and groups of relatives of patients suffering from depression (see S1 Table). In this context, the term 'patients and relatives' refers to patients with depression.
Restricting the access to lethal suicide methods was a further implemented strategy for suicide prevention. For this purpose, suicide hot spots like bridges had been identified and preventive means had been selected like the construction of barriers at jumping sites in order to prevent individuals at risk for suicidal behavior from taking their lives. Education about the toxicity of drugs was another intervention in this context (see S1 Table). 
Overall, the intervention addresses suicide prevention and better recognition and treatment of depression. Specific interventions for suicidal behaviour outside of the treatment of depression were not implemented. Nevertheless, the intervention also includes emergency cards containing information where to seek help if one has thoughts about suicide. 
The OSPI-Europe research project was executed in accordance with the principles laid down in the Helsinki declaration (2000). The OSPI-Europe interventions took place in Germany, Hungary, Ireland and Portugal. Each country had an intervention and a comparison/control site. Each of the four research teams sought ethical review and gained approval from the relevant bodies in each country: the Ethics Commission of the Medical Faculty, University of Leipzig, Germany (refs. 248-2007 and 140-2009-06072009); Semmelweis University Regional and Institutional Committee of Science and Research Ethics, Hungary (ref. TUKEB 149/2009), Ethics Research Committee of the Mid-West Regional Hospital, Limerick City and County, Ireland (no reference number, letter of approval dated 25/06/2009) and Clinical Research Ethics Committee, Merlin Park University Hospital, Galway City and County, Ireland (ref. C.A. 271); and the Ethical Committee of the Faculty of Medical Sciences, New University of Lisbon, Portugal (ref. CE/DP/7-2009). For the assessment of suicide attempts through patient records (Hungary, Portugal, partly Germany) or a routine procedure (Ireland) neither written nor verbal consent of patients was obtained. In case of interview participation, written informed consent was not obtained in order to not overwhelm the patients after suicide attempt with information and documentation. Informed verbal consent was obtained at the beginning of the interview by trained staff. A completed interview protocol functioned as documentation of participant consent. The ethics committee of each of the participating intervention regions approved this procedure prior to initiating the study.

Sample
In each of the four selected European countries in this analysis (Ireland, Portugal, Germany and Hungary) an intervention and control region was chosen. Selection was based on population size (having at least 150,000 inhabitants in each geographical area; see Table 2) and the presence of regional partners interested in cooperating within OSPI-Europe. Selection of control regions was guided by the aim to match the intervention regions concerning population size. Additional requirements were that no previous suicide prevention or depression awareness programme had taken place and that the local stakeholders/researchers were interested in participating in the project.
[…]

Study regions and time spans
The exact time spans for the intervention activities were as follows:
·	Leipzig, Germany: June 2009 – March 2011;
·	Miskolc, Hungary: January 2010 – June 2011;
·	Amadora, Portugal: April 2010 – September 2011;
·	Limerick, Ireland: January 2010 – June 2011.”	
Participants	6	(a) Cohort study—Give the eligibility criteria, and the sources and methods of selection of participants. Describe methods of follow-up
Case-control study—Give the eligibility criteria, and the sources and methods of case ascertainment and control selection. Give the rationale for the choice of cases and controls
Cross-sectional study—Give the eligibility criteria, and the sources and methods of selection of participants	15-17	“The primary outcome consisted of suicidal acts: the sum of completed suicides and attempted suicides. 
Whereas completed suicides were defined according to the ICD-10 codes [21] for intentional self-harm as an external cause of morbidity and mortality (X60-X84), with the corresponding data being provided by the national official statistical offices, attempted suicide (in the sense of a secondary outcome) was defined as “an act with non-fatal outcome, in which an individual deliberately initiates a non-habitual behaviour that, without intervention from others, will cause self-harm, or deliberately ingests a substance in excess of the prescribed or generally recognised therapeutic dosage, and which is aimed at realizing changes which the subject desired via the actual or expected physical consequences” [22]. In this context it has to be emphasized that repeated suicide attempts conducted by the same persons during the study period had not been excluded because the number of suicidal acts had been the focus of analyses, not the number of persons committing them.  
Habitual intentional self-harm is excluded by this definition. Data on attempted suicides were collected for the intervention as well as the control regions together with a given set of core variables (e.g. age, gender, region, suicide method). A standardised questionnaire for the assessment of data and a codebook containing the variables for the registration of suicide attempts were used by all partners to ensure comparability in data acquisition. Unclear cases which could not instantly be classified as a suicidal act or any other behaviour, for example habitual deliberate self-harm, were pooled. These cases were then categorised by an internal expert who was blinded regarding the timepoint and region of the event. 
The procedures for the assessment of attempted suicides differed between countries, but care was taken to assure consistency of data assessment procedures over time. In Hungary and Portugal and two of four German centres, all admissions to hospitals because of suicide attempts were assessed by using retrospective analysis of patient records. In Germany, in two further participating centres the data were assessed following a prospective design via personal interviews by trained staff. For Ireland, data of patients with a suicide attempt presenting to a hospital are routinely gathered by trained data registration officers applying standard methods for case ascertainment and definition, processed in the National Self-Harm Registry Ireland (NSHRI). From this registry, the required data could be retrieved. Levels of agreement between the data registration officers in terms of case ascertainment were high (Kappa = 0.97) [23]. 
Highly lethal suicide attempts represented another secondary outcome and implied the exclusion of the lower-risk suicide methods “Intentional drug overdose” and “use of sharp objects” which could be shown to have low case fatality ratios (1.8%) [24]. 
Thus, more lethal suicide attempts included the following suicide methods: Hanging (X70), drowning (X71), firearms (X72-X75), jumping (X80), moving objects (X81,X82), other suicide methods (X76, X77, X79, X83, X84).
If several methods were applied, the most lethal suicide method according to previous findings [25] was classified as the primary suicide method and entered into subsequent analyses.”	
		(b) Cohort study—For matched studies, give matching criteria and number of exposed and unexposed
Case-control study—For matched studies, give matching criteria and the number of controls per case		Not applicable.	
Variables	7	Clearly define all outcomes, exposures, predictors, potential confounders, and effect modifiers. Give diagnostic criteria, if applicable	15-17	See comment 6a. 	
Data sources/ measurement	8*	 For each variable of interest, give sources of data and details of methods of assessment (measurement). Describe comparability of assessment methods if there is more than one group	15-17	See comment 6a.	
Bias	9	Describe any efforts to address potential sources of bias	16	“A standardised questionnaire for the assessment of data and a codebook containing the variables for the registration of suicide attempts were used by all partners to ensure comparability in data acquisition. Unclear cases which could not instantly be classified as a suicidal act or any other behaviour, for example habitual deliberate self-harm, were pooled. These cases were then categorised by an internal expert who was blinded regarding the timepoint and region of the event.”	
Study size	10	Explain how the study size was arrived at	18	“Based on the effect sizes resulting from the Nuremberg Alliance against Depression [5], a sample size estimation was conducted a-priori to rolling out the OSPI-Europe intervention. With the level of significance () set at 0.10 (two-tailed testing) and the required power (1-) set at 0.80, assuming a decrease in suicidal acts compared to baseline of 24.8% in the intervention region and of 0 in the control region, a population of 119,071 subjects in the intervention and the control regions of each country would be necessary to observe statistically significant change.”	

Quantitative variables	11	Explain how quantitative variables were handled in the analyses. If applicable, describe which groupings were chosen and why	18	“For calculations, the absolute numbers of suicidal acts were preferred over rates because they are more informative and because demographic data revealed that there were only minor differences in the changes in the number of the population in the intervention and corresponding control regions between 2008 and 2011 (Germany: delta = -0.56%; Hungary: delta = 0.96%; Ireland: delta = 3.50%; Portugal: delta = 3.01%). Changes of the population in the OSPI intervention and control regions in the years 2008-2011 (stratified for gender) are summarized in S2 Table, the corresponding differences between OSPI intervention and control regions in S3 Table. These minor changes were corrected by a loading factor adjusting for changes of gender-specific population figures in the intervention regions (see S4 Table). Absolute numbers also reflect better the differences in the size of populations in the different cities. In addition, rates are not comparable between the cities because they differed concerning the completeness with which suicide attempts were assessed.”	
Statistical methods	12	(a) Describe all statistical methods, including those used to control for confounding	19	“Concerning the primary outcome  2 tests for two-by-two tables, with the row variable being “region” and the column variable being “time” (1=baseline; 2=arithmetic means for the two years after onset of the awareness campaign) were calculated.
Moreover, comparison of changes in the frequency of suicidal acts in the intervention versus control region was performed by using the Cochran-Mantel-Haenszel procedure, with the variable “region” being used as a stratification variable. Homogeneity of odds ratios across countries was tested by using the Breslow-Day test. 
As additional analyses 2 tests for two-by-three tables with the row variable being “region” and the column variable being “project year” (baseline, first year after start of intervention, second year after start of intervention) were performed in order to evaluate effects of the awareness campaign on the outcome variables. These analyses are presented in several tables (S5 Table, S6 Table and S7 Table).   
We used SPSS for Windows (version 20.0) for statistical analyses. 
The significance level was set at  = 0.10 (two-sided). This significance level was selected because it was essential not to miss relevant effects in view of a low rate of suicidal acts associated with a high risk that clinically important effects are overlooked.”	
		(b) Describe any methods used to examine subgroups and interactions		see above	
		(c) Explain how missing data were addressed		Not applicable. 	
		(d) Cohort study—If applicable, explain how loss to follow-up was addressed
Case-control study—If applicable, explain how matching of cases and controls was addressed
Cross-sectional study—If applicable, describe analytical methods taking account of sampling strategy		Not applicable. 	
		(e) Describe any sensitivity analyses		Not applicable.  	
Results	
Participants	13*	(a) Report numbers of individuals at each stage of study—eg numbers potentially eligible, examined for eligibility, confirmed eligible, included in the study, completing follow-up, and analysed	Supplementary Table 5	see Supplemental Table 5.	
		(b) Give reasons for non-participation at each stage		Not applicable.	
		(c) Consider use of a flow diagram		Not necessary.	
Descriptive data	14*	(a) Give characteristics of study participants (eg demographic, clinical, social) and information on exposures and potential confounders		Not applicable since suicidal acts had been analyzed.	
		(b) Indicate number of participants with missing data for each variable of interest		Not applicable since suicidal acts had been analyzed. 	
		(c) Cohort study—Summarise follow-up time (eg, average and total amount)	13-14	See Study regions and time spans 	
Outcome data	15*	Cohort study—Report numbers of outcome events or summary measures over time	Table 3	The number of suicidal acts is reported in Table 3. 	
		Case-control study—Report numbers in each exposure category, or summary measures of exposure			
		Cross-sectional study—Report numbers of outcome events or summary measures			
Main results	16	(a) Give unadjusted estimates and, if applicable, confounder-adjusted estimates and their precision (eg, 95% confidence interval). Make clear which confounders were adjusted for and why they were included		Not applicable. 	
		(b) Report category boundaries when continuous variables were categorized		Done. 	
		(c) If relevant, consider translating estimates of relative risk into absolute risk for a meaningful time period		Not relevant. 	

Other analyses	17	Report other analyses done—e.g. analyses of subgroups and interactions, and sensitivity analyses	Supplementary Tables 4-7	This was done (see in particular the Supplementary Tables 4-7).	
Discussion	
Key results	18	Summarise key results with reference to study objectives	26-30	“Our main hypothesis was not confirmed: when aggregating suicidal acts from all four intervention regions, suicidal acts did not show a statistically significant reduction compared to a one-year baseline and the control regions. However, intervention effects differed significantly between countries. At country level, a statistically non-significant effect on suicidal acts was found in Germany (intervention region: - 26; control region: + 16) and a numerically and statistically significant effect in Portugal (intervention region: - 38, control region: + 25). In Hungary, although rates decreased in both intervention and control area, no specific positive effect of the intervention was seen and even a significant effect in the opposite direction was seen in Ireland (intervention region: + 30, control region: - 65). 
The significant country differences concerning intervention effects point to the relevance of contextual factors as well as differences in the implementation process. Several external and mostly unforeseeable events which likely interfered with the OSPI-Europe interventions were identified by the OSPI-Europe process analysis (see S1 File): 
-	In Germany, the national football goalkeeper Robert Enke, who had a depressive disorder, died by railway suicide in November 2009. His death was followed by extensive media coverage, including the nationwide broadcasting of the public funeral, which was held in a large football stadium. This resulted in copycat effects [26,27], with a long-term increase of railway suicides (from 2.30 per day in the two years before Enke´s suicide to 2.73 per day in the two years after) [26]. The suicide occurred in between the two general population surveys performed within OSPI-Europe, so that the effects of media reporting on knowledge and attitudes in the general population can be estimated. As reported by Kohls et al. [19], the personal stigma decreased considerably and the value of professional help increased remarkably in Germany - in both, the intervention and control region [19], allowing the assumption that the long-lasting, intensive and in many cases sensible and informative media coverage after the suicide of Robert Enke functioned as a large overall depression awareness campaign which overshadowed possible effects of the local activities in Leipzig. 
-	In Hungary, a serious flood in spring 2010 in the intervention region during the first intervention year, as well as national elections for parliament in April and for local government in October 2010, were identified as events that strongly interfered with the intervention. The flood and the elections were dominating media reporting and made it difficult for the public awareness campaign in the intervention region to get noticed. The general population survey performed within OSPI-Europe revealed that in the intervention region in Hungary only 8.6 % of the people had noticed the intervention activities, a number which is lower than that observed in the other intervention regions (Germany: 25.8 %, Portugal: 23.6 %, Ireland: 11.2 % [19]). The process evaluation furthermore revealed that these events also impeded other intervention activities, e.g. the police was not allowed to participate in gatekeeper training, due to the need to respond to the flood and its aftermath (see S1 File). In line with these findings, there were no significant changes in negative attitudes toward depression in the sense of personal stigmatisation in the Hungarian intervention and control regions [19]. 
-	In Ireland, respondents in the process evaluation reported that the impact of the economic recession and subsequent austerity may have had a particularly negative impact on people residing in the intervention region as the region's single largest employer closed down in 2009. As a result, the intervention region, Limerick City, became the unemployment 'blackspot' [28] of the country. In 2011 it had the highest unemployment rate in the country (29%) [29]. This may have had consequences concerning access to care for people with depression and other mental disorders. 
-	In Portugal, the economic crisis hit both the intervention and the control region. 
One relevant factor explaining the fact that an intervention effect was noted for Portugal but not for any other sites could be that the percentage of individuals having self-reported experience with depression, deliberate self-harm and suicides in relatives was the highest (66%) in Portugal in comparison to Germany, Hungary and Ireland [19]. Thus, the public alertness to the target messages on the depression-related campaign seems to have been highest in Portugal and to have contributed to a significant effect of the multi-level intervention program regarding suicidal acts. 
Another possible bias has to be taken into account when discussing findings from such complex community based interventions. The intervention measures such as the training of professionals and the public awareness campaign can induce a bias by improving the recognition of suicidal acts in the intervention regions. The result can be an artificial increase in the rate of suicidal behaviour. The results of the previous Nuremberg Alliance Against Depression have pointed in such a direction: the intervention effects were smaller for suicidal behaviours using overdoses with drugs than for more lethal suicidal methods. Possibly, in the intervention region more intoxications were recognized as suicidal behaviour due to the trainings and the public relation campaign. For more lethal methods this bias can be expected to be lower because such suicidal behaviour is recognized in a more reliable manner [5]. In the present study, sub-analyses of aggregated data over all four countries did not reveal more pronounced intervention effects for more lethal suicide methods. For Ireland, however, such a recognition bias offers a possible explanation for the finding of increased suicidal acts in the intervention compared to the control region. This bias might have been large in Ireland because 66.10 % of policemen and nearly all general practitioners (GP) participated in the trainings whereas the corresponding rate in Germany was clearly lower (10.36% of policemen, 10.45% of GP) [20]. The chance that attempted or completed suicides are recognized as such by policemen as well as GP and are entered in the statistics might have increased in the intervention compared to the control region in Ireland.”
	
Limitations	19	Discuss limitations of the study, taking into account sources of potential bias or imprecision. Discuss both direction and magnitude of any potential bias	32-33	“As limitations the following points have to be mentioned: 
1) The low statistical power due to the population size in the intervention and control regions and the low base rate for suicidal behaviour result in the risk that relevant clinical effects have been overlooked. 
2) Suicidal acts diagnosed and treated outside of hospital settings were not assessed due to practical reasons. Consequently, there was not a complete assessment of attempted suicides. The impact of this limitation on the results is limited by the fact that not absolute numbers of suicidal acts were taken into account but changes over time. Care was taken to keep the assessment of attempted suicides stable over time. 
3) The intervention and respective control regions were different in size and sociodemographic structure as well as regional context. Since not baseline differences but changes over time were considered in the primary outcome evaluation, the risk of a bias introduced by these differences is limited but cannot definitively be excluded.  
4) A stratification of analyses by a financial measure and baseline differences in terms of several socio-demographic variables like age, gender, socio-economic status, and civil status would have been very interesting but was not feasible due to the limited absolute numbers of suicidal acts in the selected intervention and control regions.
5) The duration of the intervention period (1.5 years), which was significantly shorter compared to the initial implementation of the 4-level suicide prevention programme in Nuremberg, might not have been sufficient for some intervention effects to become visible.”	
Interpretation	20	Give a cautious overall interpretation of results considering objectives, limitations, multiplicity of analyses, results from similar studies, and other relevant evidence	33-34	“In summary, an optimized version of the 4-level intervention concept targeting depression and suicidal behavior was implemented in four regions in four different countries with mixed results. When aggregating data across countries, no significant effect of the multi-level suicide prevention programme OSPI-Europe was found. Therefore, the first hypothesis was not confirmed. However, effects differed significantly between countries. At the country level, a significant intervention effect on suicidal acts in the expected direction was found in Portugal but not in the other countries. Contextual factors, provided by the process evaluation and intermediate outcomes, have to be taken into account when interpreting these findings. They deliver plausible albeit only post-hoc explanations for the country differences concerning the preventive effects of the intervention on suicidal behavior.”	
Generalisability	21	Discuss the generalisability (external validity) of the study results	32	We emphasized in the Discussion section that the strengths of this study include the multinational, standardised procedure, the multifaceted intervention and the assessment of a large amount of secondary and process analysis data, thus enhancing the external validity of the findings.  	
Other information		
Funding	22	Give the source of funding and the role of the funders for the present study and, if applicable, for the original study on which the present article is based	31	We provided this information in the “Financial Disclosure Statement” as follows: 
“The research leading to these results has received funding from the European Commission within the Seventh Framework Programme (FP7/2007-2013) under Grant Agreement N° 223138. The sponsors of the study had no role in the study design, in the collection, analysis, and interpretation of data, in the writing of the report, and in the decision to submit the paper for publication. All authors had full access to the data and take responsibility for the contents of the study and the decision to proceed to publication. The corresponding author (UH) hereby confirms that he had full access to all the data in the study and had final responsibility for the decision to submit the manuscript for publication. The dataset is available on request from the corresponding author.” 	
*Give information separately for cases and controls in case-control studies and, if applicable, for exposed and unexposed groups in cohort and cross-sectional studies.

Note: An Explanation and Elaboration article discusses each checklist item and gives methodological background and published examples of transparent reporting. The STROBE checklist is best used in conjunction with this article (freely available on the Web sites of PLoS Medicine at http://www.plosmedicine.org/, Annals of Internal Medicine at http://www.annals.org/, and Epidemiology at http://www.epidem.com/). Information on the STROBE Initiative is available at www.strobe-statement.org.
