# Supplementary material for: Prevention of suicidal behaviour: Results of a controlled community-based intervention study in four European countries
Source: PLoS One. 2019 Nov 11;14(11):e0224602. doi: 10.1371/journal.pone.0224602 (PMC6844461; doi:10.1371/journal.pone.0224602)
Supplement: S1 Table — (RTF) [file pone.0224602.s002.rtf]

S1 Table. Changes of population in the OSPI-Europe intervention and control regions (2008-2011). 
Region	2008	2011	Dif-ference	Percental change	IR ver-sus CR Z(p)	Cohen's d [95% CI]	
Total population	
Intervention regions					Z=-0.58 (p=0.56)	-0.43	
						[-1.77; 1.03]	
-	Portugal	172,110	175,136	+3,026	+1.76%			
-	Germany	515,469	510,043	-5,426	-1.05%			
-	Ireland	188,299	191,809	+3,510	+1.86%			
-	Hungary	170,234	164,973	-5,261	-3.09%			
-	All four regions	1,046,112	1,041,961	-4,151	-0.40%			
Control regions							
-	Portugal	166,103	174,030	+7,927	+4.77%			
-	Germany	230,047	228,910	-1,137	-0.49%			
-	Ireland	237,898	250,653	+12,755	+5.36%			
-	Hungary	169,030	162,183	-6,847	-4.05%			
-	All four regions	803,078	815,776	+12,698	+1.58%			
Male population	
Intervention regions					Z=-0.58 (p=0.56)	-0.54	
						[-1.88; 0.93]	
-	Portugal	82,038	82,342	+304	+0.37%			
-	Germany	250,018	245,137	-4,881	-1.95%			
-	Ireland	94,563	95,815	+1,252	+1.32%			
-	Hungary	78,569	76,382	-2,187	-2.78%			
-	All four regions	505,188	499,676	-5,512	-1.09%			
Control regions							
-	Portugal	80,229	82,496	+2,267	+2.83%			
-	Germany	111,199	111,160	-39	-0.04%			
-	Ireland	118,763	124,758	+5,995	+5.05%			
-	Hungary	77,425	74,244	-3,181	-4.11%			
-	All four regions	387,616	392,658	5,042	+1.30%			
Female population	
Intervention regions					Z=-0.29 (p=0.77)	-0.33	
						[-1.68; 1.11]	
-	Portugal	90,072	92,794	+2,722	+3.02%			
-	Germany	265,451	264,906	-545	-0.21%			
-	Ireland	93,736	95,994	+2,258	+2.41%			
-	Hungary	91,665	88,591	-3,074	-3.35%			
-	All four regions	540,924	542,285	+1,361	+0.25%			
Control regions							
-	Portugal	85,874	91,534	+5,660	+6.59%			
-	Germany	118,848	117,750	-1,098	-0.92%			
-	Ireland	119,135	125,895	+6,760	+5.67%			
-	Hungary	91,605	87,939	-3,666	-4.00%			
-	All four regions	415,462	423,118	+7,656	+1.84%			
CI, confidence interval; CR, control region; IR, intervention region; OSPI-Europe, “Optimising Suicide Prevention programmes and their Implementation in Europe” funded by the European Union, 7th Framework Programme; p, significance level. 
The Z value refers to Mann-Whitney U tests for the comparison of intervention regions and control regions regarding the percentual change of population figures between 2008 and 2011.
Figures for Portugal sites are based on data from the Statistical Office of Portugal, figures for the intervention site in Germany on data from the local Information Service, figures for the control site are from the regional Statistical Office, figures for sites in Hungary are from the Hungarian Statistical Office. Figures for Ireland are based on regional estimates as exact figures for 2008 were not available.     
