# Supplementary material for: Prevention of suicidal behaviour: Results of a controlled community-based intervention study in four European countries
Source: PLoS One. 2019 Nov 11;14(11):e0224602. doi: 10.1371/journal.pone.0224602 (PMC6844461; doi:10.1371/journal.pone.0224602)
Supplement: S2 Table — (RTF) [file pone.0224602.s003.rtf]

S2 Table. Differences between OSPI-Europe intervention and control regions regarding population change between 2008 and 2011. 
Country	Total population
Ä	Male population
Ä	Female population
Ä	
Germany 	-0.56%	-1.91%	0.71%	
Hungary 	0.96%	1.33%	0.65%	
Ireland 	3.50%	3.72%	3.27%	
Portugal 	3.01%	2.46%	3.57%	
All four countries (interven-tion regions versus control regions)	1.98%	2.39%	1.59%	
OSPI-Europe, “Optimising Suicide Prevention programmes and their Implementation in Europe” funded by the European Union, 7th Framework Programme. 
The symbol Ä represents the difference between an OSPI-Europe intervention region and the assigned control region regarding changes of the corresponding populations between 2008 and 2011. 
