# Supplementary material for: Prevention of suicidal behaviour: Results of a controlled community-based intervention study in four European countries
Source: PLoS One. 2019 Nov 11;14(11):e0224602. doi: 10.1371/journal.pone.0224602 (PMC6844461; doi:10.1371/journal.pone.0224602)
Supplement: S3 Table — (RTF) [file pone.0224602.s004.rtf]

S3 Table. Number of suicidal acts after adjusting for changes of gender-specific population figures in the intervention regions. 
Coun-try	Region	Project year	Popu-lation figure	Frequency of suicidal acts	Adjusted population
figure	Adjusted number of suicidal acts	
Males	
Ger-many	Control region 	Baseline	111,199	89	111,199	89	
		Year of inter-vention	111,848	103	111,968 (251,748/ 250,018* 111,199)	103 
(111,968/ 111,848*103)	
		Follow-up	112,537	70	112,762 (253,533/ 250,018* 111,199)	70 
(112,762/ 112,537*70)	
	Inter-vention region
	Baseline	250,018	231	250,018	231	
		Year of inter-vention	251,748	222	251,748 (+0.69%)	222	
		Follow-up	253,533	206	253,533 (+1.41%)	206	
Hun-gary	Control region 
	Baseline	77,425	74	77,425	74	
		Year of inter-vention	77,653	102	76,901 (78,037/78,569* 77,425)	101 (-1) (76,901/ 77,653*102)	
		Follow-up	77,828	95	76,309 (77,437/78,569* 77,425)	93 (-2)  (76,309/ 77,828*95)	
	Inter-vention region 
	Baseline	78,569	150	78,569	150	
		Year of inter-vention	78,037	131	78,037 (-0.68%)	131	
		Follow-up	77,437	124	77,437 (-1.44%)	124	
Ireland	Control region 	Baseline	118,763	340	118,763	340	
		Year of inter-vention	119,932	315	120,168 (95,682/94,563* 118,763)	316 (+1) (120,168/ 119,932*315)	
		Follow-up	120,043	276	120,487 (95,936/94,563* 118,763)	277 (+1) (120,487/ 120,043*276)	
	Inter-vention region 	Baseline	94,563	356	94,563	356	
		Year of inter-vention	95,682	416	95,682 (+1.18%)	416	
		Follow-up	95,936	337	95,936 (+1.45%)	337	
Portu-gal	Control region	Baseline	80,229	74	80,229	74	
		Year of inter-vention	80,134	68	79,583 (81,377/82,038* 80,229)	68 (79,583/ 80,134*68)	
		Follow-up	79,978	77	78,871 (80,649/82,038* 80,229)	76 (-1)  (78,871/ 79,978*77)	
	Inter-vention region 	Baseline	82,038	106	82,038	106	
		Year of inter-vention	81,377	82	81,377 (-0.81%)	82	
		Follow-up	80,649	70	80,649 (-1.69%)	70	
Females	
Ger-many	Control region 	Baseline	118,848	91	118,848	91	
		Year of inter-vention	118,608	124	119,593 (267,114/ 265,451* 118,848)	125 (+1) (119,593/ 118,608*124)	
		Follow-up	118,988	93	120,594 (269,350/ 265,451* 118,848)	94 (+1) (120,594/ 118,988*93)	
	Inter-vention	Baseline	265,451	258	265,451	258	
		Year of inter-vention	267,114	239	267,114 (+0.63%)	239	
		Follow-up	269,350	251	269,350 (+1.47%)	251	
Hun gary	Control region 
	Baseline	91,605	130	91,605	130	
		Year of inter- vention	92,060	102	91,129 (91,189/91,665* 91,605)	101 (-1) (91,129/ 92,060*102)	
		Follow-up	92,457	75	90,579 (90,638/91,665* 91,605)	73 (-2)  (90,579/ 92,457*75)	
	Inter- vention region 
	Baseline	91,665	130	91,665	130	
		Year of inter- vention	91,189	127	91,189 (-0.52%)	127	
		Follow-up	90,638	101	90,638 (-1.12%)	101	
Ireland	Control region 	Baseline	119,135	337	119,135	337	
		Year of inter- vention	121,327	326	120,661 (94,937/93,736* 119,135)	324 (-2) (120,661/ 121,327*326)	
		Follow-up	120,596	307	120,792 (95,040/93,736* 119,135)	307 
(120,792/ 120,596*307)	
	Inter-vention region 
	Baseline	93,736	381	93,736	381	
		Year of inter- vention	94,937	458	94,937 (+1.28%)	458	
		Follow-up	95,040	323	95,040 (+1.39%)	323	
Portu gal	Control region 
	Baseline	85,874	148	85,874	148	
		Year of inter- vention	85,857	185	85,282 (89,451/90,072* 85,874)	184 (-1) (85,282/ 85,857*185)	
		Follow-up	85,780	168	84,686 (88,826/90,072* 85,874)	166 (-2) (84,686/ 85,780*168)	
	Inter- vention region 	Baseline	90,072	167	90,072	167	
		Year of inter- vention	89,451	165	89,451 (-0.69%)	165	
		Follow-up	88,826	153	88,826 (-1.38%)	153	
Missing values regarding gender (in all cases suicide attempts)	
Ger many	Inter- vention region 	Baseline		2			
		Year of inter- vention		3			
		Follow-up		8			
