# Supplementary material for: Prevention of suicidal behaviour: Results of a controlled community-based intervention study in four European countries
Source: PLoS One. 2019 Nov 11;14(11):e0224602. doi: 10.1371/journal.pone.0224602 (PMC6844461; doi:10.1371/journal.pone.0224602)
Supplement: S4 Table — (RTF) [file pone.0224602.s005.rtf]

S4 Table. Number of suicidal acts stratified for project year, region and country. 
Region	Baseline	First year after the start of the intervention	Second year after the start of the intervention	pa	
Unweighted data	
All four countries	
- Intervention region	1,781	1,843 (+3.48%)	1,573 (-11.68%)	0.86	
- Control region	1,283	1,325 (+3.27%)	1,161 (-9.51%)		
Germany 	
- Intervention region	491	464 (-5.50%)	465 (-5.30%)	0.01	
- Control region	180	227 (+26.11%)	163 (-9.44%)		
Hungary	
- Intervention region	280	258 (-7.86%)	225 (-19.64%)	0.82	
- Control region	204	204 (±0%)	170 (-16.67%)		
Ireland	
- Intervention region	737	874 (+18.59%)	660 (-10.45%)	0.0051	
- Control region	677	641 (-5.32%)	583 (-13.88%)		
Portugal	
- Intervention region	273	247 (-9.52%)	223 (-18.32%)	0.05	
- Control region	222	253 (+13.96%)	245 (+10.36%)		
Data after adjustment for changes of gender-specific population figures in the intervention regions	
All four countries	
- Intervention region	1,781	1,843 (+3.48%)	1,573 (-11.68%)	0.89	
- Control region	1,283	1,322 (+3.04%)	1,156 (-9.90%)		
Germany	
- Intervention region	491	464 (-5.50%)	465 (-5.30%)	0.0091	
- Control region	180	228 (+26.67%)	164 (-8.89%)		
Hungary	
- Intervention region	280	258 (-7.86%)	225 (-19.64%)	0.85	
- Control region	204	202 (-0.98%)	166 (-18.63%)		
Ireland	
- Intervention region	737	874 (+18.59%)	660 (-10.45%)	0.0051	
- Control region	677	640 (-5.47%)	584 (-13.74%)		
Portugal	
- Intervention region	273	247 (-9.52%)	223 (-18.32%)	0.06	
- Control region	222	252 (+13.51%)	242 (+9.01%)		
p, p value. Percentages are related to changes of the baseline values. 
a The p values (two-tailed testing) refer to the results of 2 tests for two-by-three tables, with the row variable being “region” and the column variable being “year”. 
