# Supplementary material for: Prevention of suicidal behaviour: Results of a controlled community-based intervention study in four European countries
Source: PLoS One. 2019 Nov 11;14(11):e0224602. doi: 10.1371/journal.pone.0224602 (PMC6844461; doi:10.1371/journal.pone.0224602)
Supplement: S5 Table — (RTF) [file pone.0224602.s006.rtf]

S5 Table. Number of attempted suicides stratified for project year, region and country. 
Region	Baseline	First year after the start of the intervention	Second year after the start of the intervention	pa	
Unweighted data	
All four countries	
- Intervention region	1,643	1,671 (+1.70%)	1,419 (-13.63%)	0.91	
- Control region	1,195	1,210 (+1.26%)	1,051 (-12.05%)		
Germany	
- Intervention region	418	390 (-6.70%)	400 (-4.31%)	<0.01	
- Control region	155	207 (+33.55%)	148 (-4.52%)		
Hungary	
- Intervention region	230	206 (-10.43%)	186 (-19.13%)	0.59	
- Control region	169	160 (-5.33%)	123 (-27.22%)		
Ireland	
- Intervention region	733	838 (+14.32%)	631 (-13.92%)	<0.01	
- Control region	669	602 (-10.01%)	552 (-17.49%)		
Portugal	
- Intervention region	262	237 (-9.54%)	202 (-22.90%)	0.01	
- Control region	202	241 (+19.31%)	228 (+12.87%)		
Data after adjustment for changes of gender-specific population figures in the intervention regions	
All four countries	
- Intervention region	1,643	1,671 (+1.70%)	1,419 (-13.63%)	0.92	
- Control region	1,195	1,207 (+1.00%)	1,048 (-12.30%)		
Germany	
- Intervention region	418	390 (-6.70%)	400 (-4.31%)	<0.01	
- Control region	155	208 (+34.19%)	149 (-3.87%)		
Hungary	
- Intervention region	230	206 (-10.43%)	186 (-19.13%)	0.56	
- Control region	169	158 (-6.51%)	121 (-28.40%)		
Ireland	
- Intervention region	733	838 (+14.32%)	631 (-13.92%)	<0.01	
- Control region	669	601 (-10.16%)	553 (-17.34%)		
Portugal	
- Intervention region	262	237 (-9.54%)	202 (-22.90%)	0.02	
- Control region	202	240 (+18.81%)	225 (+11.39%)		
p, p value. Percentages are related to changes of the baseline values. 
a The p values (two-tailed testing) refer to the results of 2 tests for two-by-three tables, with the row variable being “region” and the column variable being “year”. 
