# Supplementary material for: Prevention of suicidal behaviour: Results of a controlled community-based intervention study in four European countries
Source: PLoS One. 2019 Nov 11;14(11):e0224602. doi: 10.1371/journal.pone.0224602 (PMC6844461; doi:10.1371/journal.pone.0224602)
Supplement: S6 Table — (RTF) [file pone.0224602.s007.rtf]

S6 Table. Number of completed suicides stratified for project year and region. 
Region	Baseline	First year after the start of the intervention	Second year after the start of the intervention	pa	
Unweighted data	
- All intervention regions	138	172 (+24.64%)	154 (+11.59%)	0.82	
- All control regions	88	115 (+30.68%)	110 (+25.00%)		
Data after adjustment for changes of gender-specific population figures in the intervention regions	
- All intervention regions	138	172 (+24.64%)	154 (+11.59%)	0.85	
- All control regions	88	115 (+30.68%)	109 (+23.86%)		
p, p value. Percentages are related to changes of the baseline values. 
a The p values (two-tailed testing) refer to the results of 2 tests for two-by-three tables, with the row variable being “region” and the column variable being “year”. 
