# Supplementary material for: Prevention of suicidal behaviour: Results of a controlled community-based intervention study in four European countries
Source: PLoS One. 2019 Nov 11;14(11):e0224602. doi: 10.1371/journal.pone.0224602 (PMC6844461; doi:10.1371/journal.pone.0224602)
Supplement: S7 Table — (RTF) [file pone.0224602.s008.rtf]

S7 Table OSPI-Europe Programme Theory of Change
Intervention levels	Programme theory/ anticipated mechanisms of action	Anticipated intermediate outcomes	Anticipated primary outcome	
The combination of activity at all levels of intervention will yield greater benefits than activities in individual levels alone and create local, collaborative networks/ local AGs. 	AG members share ideas and knowledge, increase reach and capacity, extend their networks and are transformed to take ownership for maintaining longer term delivery. Willingness of local partners and organisations to do something: collective goals around depression and/or suicide.	Achievement of (minimal intensity) goals across all levels.	Reduction of suicidal acts. Reduction of suicidal acts by increasing opportunities for help seeking and referral of suicidal persons (Note: these were not collected as  outcomes). 	
Level 1 GP and Level 3 CF gatekeeper training Purpose: to engage and transform GP and other gatekeeper (CFs) interest, knowledge and awareness of depression and suicide.	For implementation: High level gatekeepers recognise affinity with OSPI-Europe goals and/or target populations, recognise a knowledge gap/needs for their organisation/staff and promote or sanction delivery of training to members/staff. For impact: GPs and CF's acknowledge their lack of skills and salience of skills acquisition for their role(s)	Achievement of (minimal intensity goals). Increased awareness, improved knowledge and attitudes of level 1 and 3 gatekeepers. Changing professional behaviour/practice	Reduction of suicidal acts through better detection and management of depression and those at risk of suicide. 	
Level 3 Train the Trainer (CF training) Purpose: to ensure sustainability of training beyond the OSPI-Europe intervention period (not conducted in all intervention regions).	For implementation: High level gatekeepers recognise the importance of continuing member/staff needs and transform their participation to an 'ownership' role. Support for trainers from the OSPI-Europe team will maintain quality of trainers and content of training. For impact: Trainers take ownership and responsibility for delivery; organisational support for the recognised role of trainer and its continuation.	Trained trainers are available to deliver further training. Sustainability of TtT model.	Capacity and sustainability building. Reduction of suicidal acts through better identification and referral/ signposting of those at risk.	
Level 2 Public Awareness Purpose: to engage and transform public interest and awareness in depression. To increase mental health literacy and reduce stigmatising attitudes.	For implementation: collaborative partners will share key aims of campaigns and contribute to capacity and dissemination. For impact: Those at risk for depression or suicidal behaviour identify with, and salience of, campaign messages. Knowledge of helplines etc. will enable them to take action to seek help. Exposure to knowledge of depression will 'normalise' rather than 'stigmatise' mental health. 	Achievement of minimal intensity goals. Improved knowledge and attitudes. Reduction of stigma. Improvement in help seeking (attitudes and behaviour).	Reduction of suicidal acts by reducing stigma and thereby increasing help seeking behaviour of those 'at risk'.	
Level 4 Locally driven interventions for patients, high-risk groups, and their relatives. Purpose: increase up-take of offers of help	For implementation: engagement of emergency/hospital sector to ensure reach of target populations. For impact: At risk-groups or families 'identify' with efforts made, and trust sources of help. Positive re-enforcement/feedback from other user communities.	Increased help seeking. Increased use of self-help type supports. (Activities and outcomes not quantified at this level.)	Reduction of suicidal acts by reducing stigma and thereby increasing help seeking behaviour of those 'at risk'. Reduction of suicidal acts through complemented care.	
Developing locally based actions on restricting access to lethal means. Purpose: to identify local suicide 'hot spots' and other lethal means, e.g. medication, and to work with relevant authorities to develop solutions.	For implementation: Local knowledge of partners/stake-holders will identify hot spots. Relevant authorities will already be AG members or will be invited to join. Relevant authorities will exert influence as required to develop, resource and implement action.	Any local action to restrict access to means or increase potential for saving lives. (Activities and outcomes not quantified at this level.)	Reduction of suicidal acts through restricting access to means.	
AG, advisory group; CF, community facilitator; GP, general practitioner; OSPI-Europe, “Optimising Suicide Prevention programmes and their Implementation in Europe” funded by the European Union, 7th Framework Programme; TtT, train-the-trainer. 
