# Supplementary material for: Prevention of suicidal behaviour: Results of a controlled community-based intervention study in four European countries
Source: PLoS One. 2019 Nov 11;14(11):e0224602. doi: 10.1371/journal.pone.0224602 (PMC6844461; doi:10.1371/journal.pone.0224602)
Supplement: S8 Table — (RTF) [file pone.0224602.s009.rtf]

Supplementary Table 8: Core and optional intervention measures of OSPI-Europe
		Core measures	Optional measures	
Level 1	Target groups	General practitioners (GPs) in the sense of physicians based in the community, treating patients with minor or chronic diseases and referring patients with serious illnesses to a hospital	GPs, paediatricians, primary care nurses/public health nurses, primary care psychologists, social workers, etc.	
	Intervention measures	Trainings and workshops (with accreditation for continuous medical education (CME), Informational videos/DVDs for patients, Educational videos/DVDs for GPs)
Training of GPs was implemented by trained professionals; related to the extent of depressive disorders and suicidal behaviour, warning signs as well as risk factors for suicidality, diagnosis and therapy of depressive disorders, dealing with an acute suicidal crisis; included role-playing in order to explore suicidal behaviour in patients; duration: 4 hours (in Germany, Hungary and Ireland; 8 hours in Portugal); monitoring of training effects regarding knowledge by using three measures: the Depression Attitude Questionnaire [61], the Attitude towards Suicide Prevention Scale [62], and the Morriss Confidence Scale [63] (for details see [27]).        	Trainings and workshops for optional target groups (with accreditation for continuous medical education (CME), telephone helpline or online counselling for GPs in case of specific questions, training in cognitive behavioural therapy for psychiatric professionals	
Level 2	Target group	General public	
	Intervention measures	Flyer, poster (large and small; showing faces of individuals with a broad age spectrum; including crisis hotline numbers and hints for medical treatment of depression; displayed at large billboards, poster pillars and info towers; evidence used to develop them derived from the Nuremberg alliance against depression), campaign opening ceremony, engagement of a well-known patron, public events (of informational or active nature, e.g. penal discussion, depression day, jogging against depression), press conference	Placards, poster (medium-size and very small), brochures, press kits, informational CDs, cinema spot, newsletter, website	
Level 3	Target group	Pharmacists, priests and religious agents (main religion), policemen selected because their important role as individuals being frequently confronted with depression and suicidality in the context of their daily work activities; training implemented by trained professionals (see level 1).   
	Professional groups with a potential gatekeeper function for depressed patients, e.g. hotline professionals, prison professionals, teachers, midwifes, journalists, social workers, counselling centre workers, carers for the young and the elderly	
	Intervention measure	Train the trainer seminars, workshops, information material, media guideline for journalists	Workshops for optional target groups, including journalists	

Level 4	Target group	Patients and their relatives
	High risk groups	
	Intervention measure	Support for self-help groups for depression; emergency cards for high risk patients defined by psychiatric assessment after suicide attempt; information material	Self-help groups for relatives (bereaved); informational videos for high-risk groups; postcards	

Additional measure	Target groups	Professionals, individuals at risk for suicidal behaviour	
	Intervention measures	Identification of suicide hot spots; potentially construction of barriers at jumping sites; education about toxicity of drugs in Level 1 and 3 courses	information at hotspots (e.g. emergency telephone number)	

Notes:
61. Botega NJ, Blizard R, Wilkinson D, Mann A. General practitioners and depression – first use of the depression attitude questionnaire. Intern J Methods Psychiatr Res. 1992;4: 169–180.
62. Herron J, Ticehurst H, Appleby L, Perry A, Cordingley L. Attitudes towards suicide prevention in front-line health staff. Suicide Life Threat Behav. 2001;31: 342–347.
63. Morriss R, Gask L, Battersby L, Francheschini A, Robson M. 1999. Teaching frontline health and voluntary workers to assess and manage suicidal patients. J Affect Disord. 1999;52: 77–83.
